# Decade-long persistence of CD19 CAR T cells in B cell lymphomas

**DOI:** 10.1038/s41591-026-04578-1

**Published:** 2026-08-10

**Authors:** Luca Paruzzo, Emeline R. Chong, Elise A. Chong, Zhixuan Zheng, Puneeth Guruprasad, Daniel J. Landsburg, Sunita D. Nasta, Federico Stella, Ranjani Ramasubramanian, Vitor B. de Souza, Mohannad H. Alkhatib, Matthew Ho, Antonio Imparato, Gregory M. Chen, Don L. Siegel, Gabriela Plesa, Anlan Dai, Shane Mackey, Priyamvada Das, Peter Michener, Julie Jadlowsky, Frederic D. Bushman, Vrutti Patel, Alberto Carturan, Ellen B. Napier, Vanessa E. Gonzalez, Danuta Jarocha, Rong Xu, Bruce L. Levine, Patrizia Porazzi, Noelle Frey, David L. Porter, Jakub Svoboda, Joseph A. Fraietta, Carl H. June, Stephen J. Schuster, Marco Ruella

**Affiliations:** 1 Lymphoma Program, Abramson Cancer Center, University of Pennsylvania, Philadelphia, PA USA; 2 Center for Cellular Immunotherapies, University of Pennsylvania, Philadelphia, PA USA; 3 Department of Medicine, Division of Hematology/Oncology, Hospital of the University of Pennsylvania, Philadelphia, PA USA; 4 Department of Microbiology, Perelman School of Medicine, University of Pennsylvania, Philadelphia, PA USA; 5 Department of Pathology and Laboratory Medicine, Hospital of the University of Pennsylvania, Philadelphia, PA USA; 6 Center for Cell Therapy and Transplant, Philadelphia, PA USA

**Keywords:** Translational research, Cancer immunotherapy, B-cell lymphoma, Translational immunology

## Abstract

Chimeric antigen receptor (CAR) T cell therapy induces durable remissions in lymphoid malignancies, yet the extent and biology of long-term CAR T cell persistence in B cell lymphoma remain unclear. Here we report the persistence and characteristics of 4-1BB-costimulated anti-CD19 CAR T cells (CART19) up to 10 years after infusion in 38 patients with non-Hodgkin lymphoma. Beyond year five, the *CAR19* transgene was detectable in five of eight long-term responders (7.0–10.1 years), with three patients maintaining B cell aplasia, which is consistent with sustained functional activity. In one patient with a progression-free survival of 10.1 years, CART19 cells comprised 1.2% of circulating T cells 9.3 years after infusion. Long-term persisting CART19 cells exhibited a predominant double-negative (CD4^−^CD8^−^), effector-memory-like phenotype associated with increased aerobic metabolism and T cell activation programs. Longitudinal profiling revealed a progressive transition from CD8^+^ to double-negative CAR T cells over time. Persisting CART19 shared transcriptional features with long-term CAR T cells described in acute and chronic leukemias. T cell receptor sequencing demonstrated oligoclonal persistence at 9.3 years, with a dominant clone (70% of CART19 cells) already detectable at low frequency (<0.1%) at day 14. Lentiviral integration-site analysis identified a predominant integration within PACS1 without evidence of known drivers of CAR T cell expansion. These findings demonstrate that CART19 cells can persist for more than 10 years in lymphoma and identify phenotypic, transcriptional and clonal features associated with exceptionally long-term persistence. ClinicalTrials.gov registration: NCT02030834

## Abstract (web summary)

Analysis of longitudinal blood samples shows that CD19 CAR T cells can be detected at least 10 years after treatment in patients with lymphoma, with deeper profiling from a single patient showing that these CAR T cells have a dominant double-negative activated effector-memory-like phenotype.

## Main

Chimeric antigen receptor (CAR) T cell therapy targeting CD19 has transformed the treatment landscape of relapsed or refractory (R/R) B cell malignancies. In B cell non-Hodgkin lymphomas (B-NHLs), CD19-directed CAR T cells are now the standard of care after failure of first-line chemoimmunotherapy^1–11^. Long-term follow-up from pivotal and real-world studies shows that 3–5-year progression-free and event-free survival curves plateau at approximately 30–40%, depending on histological subtype and clinical features, suggesting that a subset of patients may achieve durable, potentially curative remissions^5,12–14^. We recently reported the 10-year clinical outcomes of CART19 in lymphoma showing a lymphoma-free survival of 32% for large B-cell lymphoma and 47% for follicular lymphoma^15^.

Multiple factors influence durable responses to CAR T cell therapy, including tumor burden, the tumor microenvironment and host immunity, but most ultimately act by constraining CAR T cell function. Consequently, CAR T cell effector function, expansion and persistence are key determinants of clinical outcome^13,16^. Mechanistic insights from chronic lymphocytic leukemia (CLL) and B cell acute lymphoblastic leukemia (B-ALL) indicate that long-term CAR T cell persistence is associated with sustained remission, with functional CAR T cells detectable up to a decade after infusion^17–20^. Long-term CAR T cell persistence has also been described beyond hematological malignancies; for example, in neuroblastoma, longer persistence of first-generation CAR T cells was associated with improved survival^21^. However, in B-NHL, the extent, durability and clinical relevance of long-term CAR T cell persistence, especially at 10 years, remain unclear. Indeed, results across studies are heterogeneous and depend on CAR construct, histological subtype and methods used to assess CAR T cell kinetics^5^^,15^^,22^^,23^.

Furthermore, the biological features of long-lived CAR T cells appear to vary across diseases and CAR constructs. In CLL, persisting CAR T cells (7.2–9.3 years after infusion) have been described as CD4^+^ and oligoclonal, whereas in pediatric B-ALL, long-term persisting CAR T cells (0.6–5 years after infusion) are double-negative (DN) (CD4^−^CD8^−^) and remain polyclonal. Yet, transcriptional profiling in leukemia has revealed that persisting CAR T cells converge on an ‘exhausted-like memory’ phenotype characterized by expression of *TIM-3*, *LAG3*, *TIGIT* and *HLA-DR*^17,18^. These findings suggest a shared trajectory of CAR T cell evolution toward a long-lived state that may support long-term tumor control. Nevertheless, in-depth phenotypic and transcriptional characterization of persisting CAR T cells has been largely limited to leukemias, where long-term persistence is more commonly observed^13,17^. Whether similar persistence and evolutionary trajectories occur in lymphoma remains unknown.

In this study, we demonstrate decade-long persistence of CD19-directed CAR T cells (CART19, now tisagenlecleucel) in adults with B-NHL treated on the single‑center trial NCT02030834 (refs. ^1^^,5^^,15^) and present an in-depth analysis. Using integrated multiomics analyses, we comprehensively characterized long-term CAR T cell persistence in lymphoma and defined the phenotypic, transcriptional and clonal features of cells that persist for up to 10 years after infusion.

## Results

### CAR T cells can persist for up to a decade in patients treated for B-NHLs

We analyzed CAR T cell persistence in longitudinal clinical samples from 38 patients with B-NHL treated with CART19 in the single-center trial NCT02030834 (refs. ^1,5,15^). Baseline patient characteristics have been reported previously. Briefly, 24 patients had large B cell lymphomas (LBCLs); 14 had follicular lymphoma (FL). Patients received 1.0–5.0 × 10^8^ CAR T cells after lymphodepleting chemotherapy. Lymphodepletion included cyclophosphamide-based (Cy-based) regimens in 21 patients, bendamustine in 16 patients and carboplatin-gemcitabine in one patient. In R/R LBCL, the best overall response (BOR) rate was 58% (14 of 24 patients), with 11 of 24 patients (46%) achieving a complete response (CR); in R/R FL, the BOR was 79% (11 of 14) and the CR was 71% (10 of 14). Any-grade cytokine release syndrome (CRS) and immune effector cell-associated neurotoxicity syndrome (ICANS) occurred in 58% and 11% of patients, respectively^1,5^. Long-term follow-up (10.1 years, range, 7.9–11.5) showed no relapses beyond 5.4 years. Further, the 10-year lymphoma-free survival was 32% (95% confidence interval (CI), 14–51) for LBCL and 47% (95% CI, 20–71) for follicular lymphoma^15^.

**Fig. 1:**
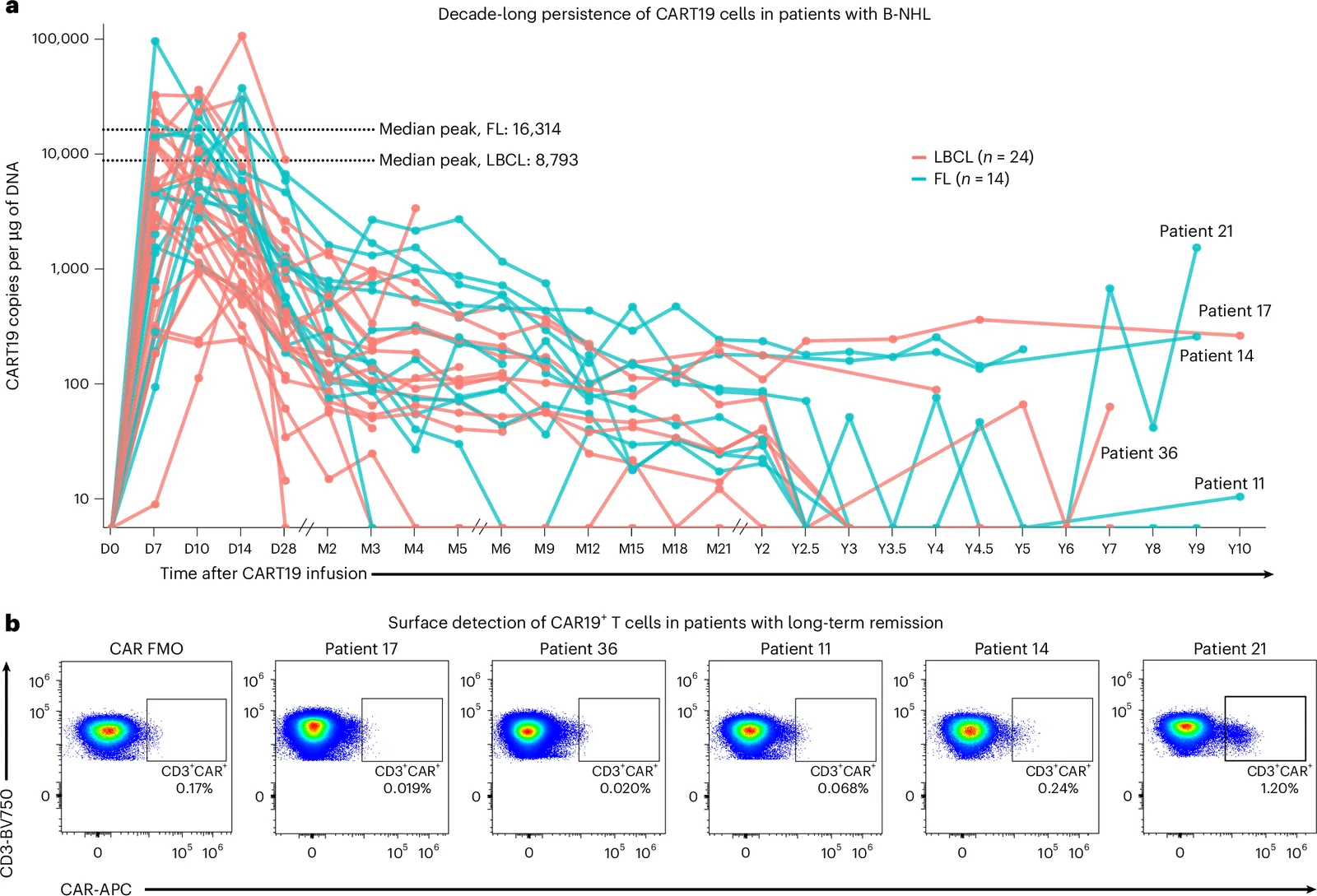
Decade-long CART19 cell persistence in patients with NHL. **a**, CART19 transgene copies over time, as measured using quantitative PCR (qPCR). The pale red lines indicate patients treated for LBCL; the light blue lines indicate patients treated for FL. The detection limit for samples collected between 2.5 and 7 years after infusion was 50 copies per µg of DNA, while the long-term persistence detection limit was five copies per µg of DNA. The dashed lines indicate the median peak value based on the disease. **b**, Flow cytometry detection of CAR T cells in patients with positive qPCR for the CAR transgene. Gating was performed using a fluorescence minus one control for the CAR antibody. Patient 21 was the only case showing a clearly distinguishable CAR^+^ population. LBCL, large B cell lymphomas; FL, follicular lymphoma; APC, allophycocyanin; BV, Brilliant Violet; D, day; M, month; Y, year.

Peak CART19 expansion occurred on day 7 in 18 patients (47.3%), on day 10 in 13 (34.2%) and on day 14 in seven (18.5%). The timing of peak expansion was similar between LBCL and FL, with most patients peaking at day 7 (50% versus 43%), followed by day 10 (33% versus 36%) and day 14 (17% versus 21%) (*P* = 0.91). The median peak transgene level was 11,096 CAR T copies per µg of DNA (interquartile range (IQR) 3,358–22,899). In LBCL (*n* = 24), the median peak was 8,793 copies per µg of DNA (IQR = 2,247–18,502), compared with 16,314 in FL (*n* = 14, IQR = 4,880–27,691; *P* = 0.26) (Fig. 1a). Transgene copies declined over time, with median levels of 141 copies per µg of DNA (*n* = 31, IQR = 88–501) at month 3, 126 (*n* = 23, IQR = 48–365) at month 6, 82 (*n* = 19, IQR = 45–168) at year 1 and 41 (*n* = 16, IQR = 28–93) at year 2.

**Extended Data Fig. 1:**
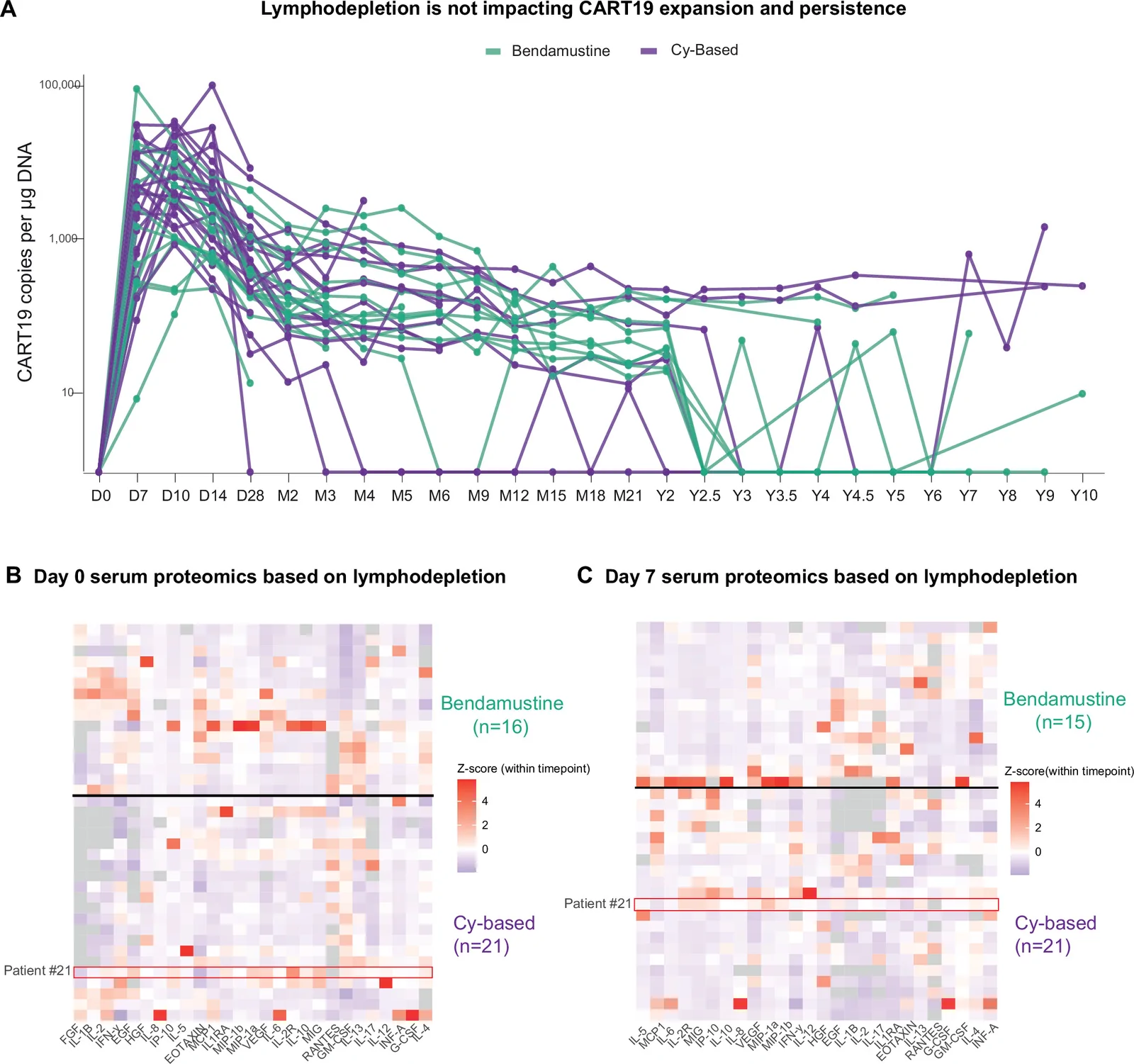
Lymphodepletion impacts on CAR+ T expansion and serum macroenvironment. (**A**) CAR^+^ T-cell expansion quantified by quantitative polymerase chain reaction (qPCR) according to lymphodepletion regimen. Sixteen patients received bendamustine-based lymphodepletion (green lines) and 21 received cyclophosphamide-based lymphodepletion (Cy-based, purple lines). One patient was excluded from this analysis because they received platinum-based lymphodepletion. (**B**) Heatmap depicting z-scores of protein concentrations measured by Luminex at day 0 post CAR T-cell infusion. Patients receiving bendamustine-based lymphodepletion are shown in the top half (*n* = 16), and those receiving cyclophosphamide-based lymphodepletion are in the bottom half (*n* = 21). Gray indicates proteins that were not quantifiable. Proteins with more than 50% missing values were excluded from the analysis. (**C**) Heatmap depicting z-scores of protein concentrations measured by Luminex at day 7 post CAR T-cell infusion. Patients receiving bendamustine-based lymphodepletion are shown in the top half (*n* = 15), and those receiving cyclophosphamide-based lymphodepletion are in the bottom half (*n* = 21). Gray indicates proteins that were not quantifiable. Proteins with more than 50% missing values were excluded from the analysis.

We next evaluated very long-term persistence. Beyond year five, 12 patients remained in remission and were defined long-term responders^15^. Samples suitable for CART19 persistence testing were available in eight of these patients. In five of eight patients (62.5%) (three FL and two LBCL), CAR T cells were still detectable using qPCR, with a median of 262 copies per µg of DNA (range, 10–1,567 copies per µg of DNA; Fig. 1a and Extended Data Table 1). We first asked whether molecular persistence corresponded to phenotypically detectable CAR T cells using multiparameter cytometry at the same or closest time point. CAR T cells were clearly detectable using flow cytometry only in patient 21 (1,567 copies per µg of DNA at the matched time point), where they comprised 1.2% of total CD3^+^ lymphocytes (Fig. 1b). All three patients with more than 200 transgene copies per µg of DNA had complete and sustained B cell aplasia, whereas the two patients with lower-level persistence showed B cell reconstitution (Extended Data Table 1). We then evaluated whether the type of lymphodepletion (Cy versus Benda-based) influenced CAR T cell expansion or long-term persistence and found no association (Supplementary Tables 1 and 2 and Extended Data Fig. 1a). Similarly, post-infusion serum cytokine levels did not differ according to lymphodepletion regimen (Extended Data Fig. 1b,c and Supplementary Table 3). Baseline B cell counts and phenotypic characteristics of the CAR T cell products were not associated with long-term outcomes, probably reflecting the limited sample size (Supplementary Figs. 1–3 and Supplementary Table 4).

Together, these data show that CD19-directed CAR T cells can remain functionally active for up to a decade in B-NHL, maintaining durable B cell aplasia in a subset of long-term responders.

### Long-term persistence in patient 21 is associated with sustained B cell aplasia and recurrent infections

To define the phenotype of persisting CAR T cells, we focused on peripheral blood CAR T cells from patient 21 at year 9.3. Patient 21 is a 68-year-old woman with R/R FL who relapsed after six lines of therapy. At enrollment, she had stage IV disease with bone marrow (<5% infiltration) and cutaneous involvement, without bulky disease. She received intravenous cyclophosphamide (300 mg m^−^^2^ daily for 4 days) as bridging therapy because of ongoing disease progression, followed by lymphodepleting chemotherapy with cyclophosphamide (300 mg m^−^^2^ daily for 4 days). At infusion, lactate dehydrogenase (LDH) was within normal limits. After CART19 infusion, the patient had no CRS or ICANS but experienced persisting long-term neutropenia likely related to concurrent clonal hematopoiesis (*TET2* and *DNMT3A* mutations; Supplementary Fig. 4). She achieved a CR at month 1 and remains in remission after 10.1 years.

**Fig. 2:**
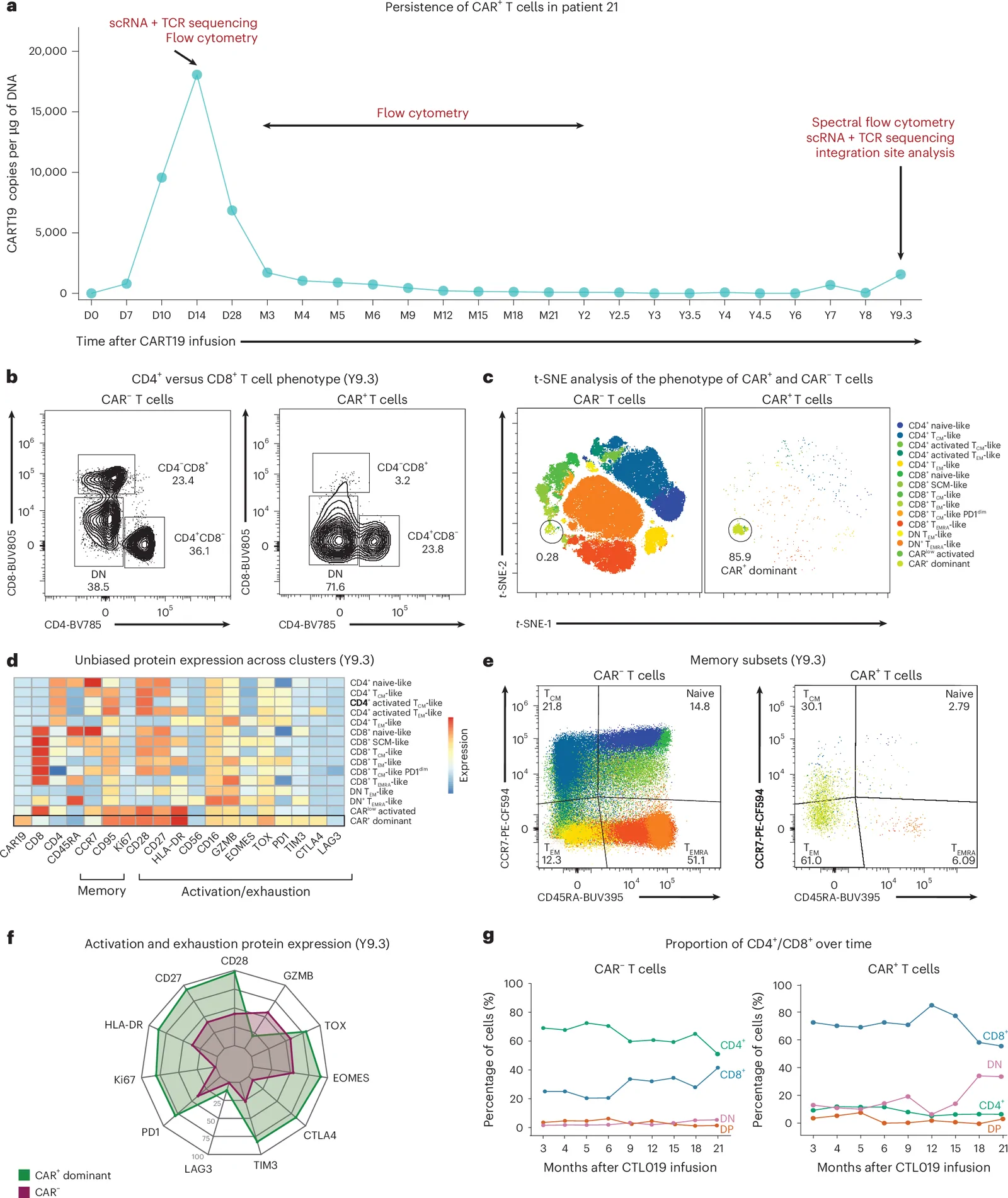
Patient 21 CAR^+^ T cells have a dominant DN effector phenotype. **a**, Schematic of the analysis performed on PBMCs from patient 21. The peak time point was identified on day 14. Long-term CAR T persistence was characterized by two distinct expansion flares, the latest occurring at year 9.3. **b**, Contour plots showing the percentage of CD4^+^, CD8^+^ and DN (CD4^−^CD8^−^) T cells within the CAR^−^ and CAR^+^ compartments. **c**, *t*-distributed stochastic neighbor embedding (*t*-SNE)-based unbiased T cell clustering derived from surface protein expression detected using multiparameter flow cytometry. CAR expression was not included as a parameter for clustering. Mapping of CAR T cells onto the identified T cell clusters shows that CAR T cells are located predominantly within a single dominant cluster. **d**, Heatmap of normalized expression (normalized mean fluorescence intensity) of selected T cell markers across clusters. The dominant CAR^+^ cluster is highlighted by the black box. **e**, Dot plots color-coded by *t*-SNE-defined clusters for CAR^−^ T cells (left) and CAR^+^ T cells (right), annotated according to memory phenotype: naive (CD45RA^+^CCR7^+^), central memory (T_CM_; CD45RA^−^CCR7^+^), effector memory (T_EM_; CD45RA^−^CCR7^−^), and T effector memory re-expressing CD45RA (T_EMRA_) (CD45RA^+^CCR7^−^). While CAR^−^ T cells are distributed across all four quadrants, CAR^+^ T cells are predominantly T_EM_, with the dominant CAR T cluster comprising nearly the entirety of the T_EM_ compartment. **f**, Radar plot representing the percentage of cells expressing the visualized markers, comparing the dominant CAR T cluster with the bulk of the CAR^−^ T cells. **g**, Percentage of CD4^+^(green), CD8^+^(blue), double positive (DP) (CD4^+^CD8^+^, orange) and DN (CD4^−^CD8^−^, purple) CAR^−^ and CAR^+^ T cells of patient 21 from month 3 to month 21. BUV, Brilliant Ultraviolet; SCM, stem cell memory T cell. Source data

CAR transgene levels peaked on day 14 (18,062 copies per µg of DNA) and declined through year 2, thereafter persisting at low levels (72–77 copies per µg of DNA) before falling below the limit of detection at year 4.5 (Fig. 2a). Notably, long-term persistence was characterized by two late expansion flares: one at year 7 (689 copies per µg of DNA) and another at year 9.3 (1,567 copies per µg of DNA), when CAR T cells represented 1.2% of total CD3^+^ T cells using flow cytometry. No infectious events or vaccinations could be clearly linked with the first flare, but at the time of the second expansion, the patient had a nasal swab positive for rhinovirus/enterovirus. CAR T cells remained functionally active after infusion, as evidenced by sustained B cell aplasia and profound hypogammaglobulinemia requiring monthly intravenous immunoglobulin (IVIG) replacement. Despite IVIG, the patient experienced recurrent infections, including persistent bronchiolitis beginning in year 9 and ongoing at last follow-up (year 10.1; Fig. 2a).

### Long-term CAR T cells have a double CD4 and CD8 negative activated effector-memory-like phenotype

**Extended Data Fig. 2:**
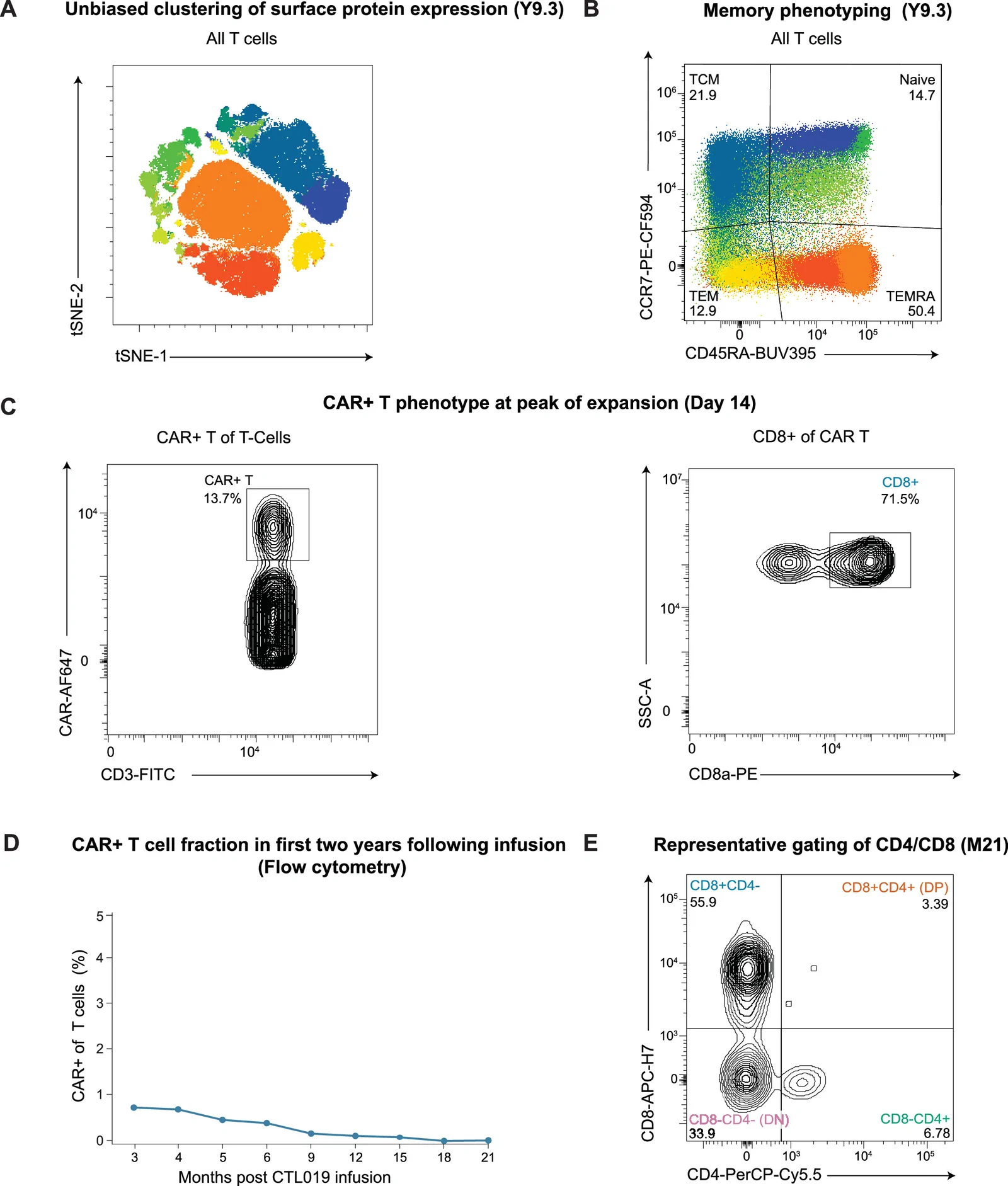
Patient #21 T-cell phenotype over time. (**A**) t-distributed Stochastic Neighbor Embedding (t-SNE)-based unbiased T-cell clustering derived from surface protein expression detected by multiparameter flow cytometry. CAR expression was not included as a parameter for clustering. (**B**) Contour plots color-coded by t-SNE-defined clusters for all T-cells annotated by memory phenotype: naïve (CD45RA^+^CCR7^+^), central memory (TCM; CD45RA^−^CCR7^+^), effector memory (TEM; CD45RA^−^CCR7^−^), and TEMRA (CD45RA^+^CCR7^−^). (**C**) Contour plots defining CAR^+^ T-cells and CD8^+^CAR^+^ T-cells at the peak time point. (**D**) CAR T percentage of total T-cells in patient #21 from Month (M) 3 to Month 21. (**E**) Representative contour plot showing the distribution of CD4^+^, CD8^+^, Double Positive (CD4^+^CD8^+^), and double negative (CD4^−^CD8^−^) CAR T in the M21 sample.

Phenotypic analysis of patient 21 revealed that long-term-persisting CAR^+^ T cells were enriched for DN (CD4^−^CD8^−^) T cells (71.6% versus 38.5%) compared with CAR^−^ T cells; CD8^+^ T cells were reduced (3.2% versus 23.4%), whereas CD4^+^ (23.8% versus 36.1%) and DP (CD4^+^CD8^+^) T cells (0.01% versus 0.41%) were similar (Fig. 2b). Unbiased clustering (excluding CAR as a feature) identified 15 T cell clusters. Notably, 86% of CAR^+^ T cells were compactly localized to a single dominant cluster (Fig. 2c). This cluster exhibited a DN (CD4^−^CD8^−^) and effector-memory-like phenotype (CD45RA^−^CCR7^−^CD95^+^). Only 0.28% of CAR^−^ T cells shared this phenotypic profile, highlighting the unique features of long-term-persisting CAR T cells. Compared with the CAR^−^ T cell compartment, this dominant CAR^+^ cluster demonstrated higher proliferation (Ki-67), activation (HLA-DR) and co-stimulatory receptor expression (CD27, CD28), together with increased immune checkpoint expression (PD-1, TIM-3, CTLA-4). Conversely, granzyme B (GZMB) expression was lower than in CAR^−^ T cells, indicating a distinct cytotoxic program in long-term persisting CAR T cells (Fig. 2c–f and Extended Data Fig. 2a,b). We next asked whether the DN CAR T cells observed at year 9.3 in patient 21 were already present at the peak of expansion (day +14; CAR transgene copies, 18,062 per µg of DNA). At the peak time point, most CAR^+^ cells were CD8^+^ CAR T cells (71.5% of total CART19; Extended Data Fig. 2c), which is consistent with expected CAR T kinetics^24^. The proportion of CAR^+^ DN T cells at peak was not associated with clinical response (Supplementary Fig. 5).

We then explored the dynamics of DN CAR T cells over time in patient 21 by analyzing flow cytometry longitudinally from month 3 through month 24. The frequency of CAR^+^ T cells declined from 0.76% of total T cells on month 3 to 0.04% on month 21, falling below the limit of reliable flow cytometry detection by month 24 (Extended Data Fig. 2d). Within the CAR^+^ compartment, the DN fraction increased from 13.4% on month 3 to 33.9% on month 21, whereas CD8^+^ CAR T cells decreased reciprocally, suggesting progressive DN enrichment over time. In contrast, DN T cells within the CAR^−^ compartment remained stable at 1–5%, indicating selective expansion of DN CAR T cells during long-term follow-up (Fig. 2g and Extended Data Fig. 2e).

Together, these data highlight the long-term emergence and persistence of DN proliferating functionally active CAR T cells that sustain ongoing remission in patient 21.

### Long-term persisting CAR T cell transcriptional analysis reveals enhanced activation and aerobic metabolism

**Fig. 3:**
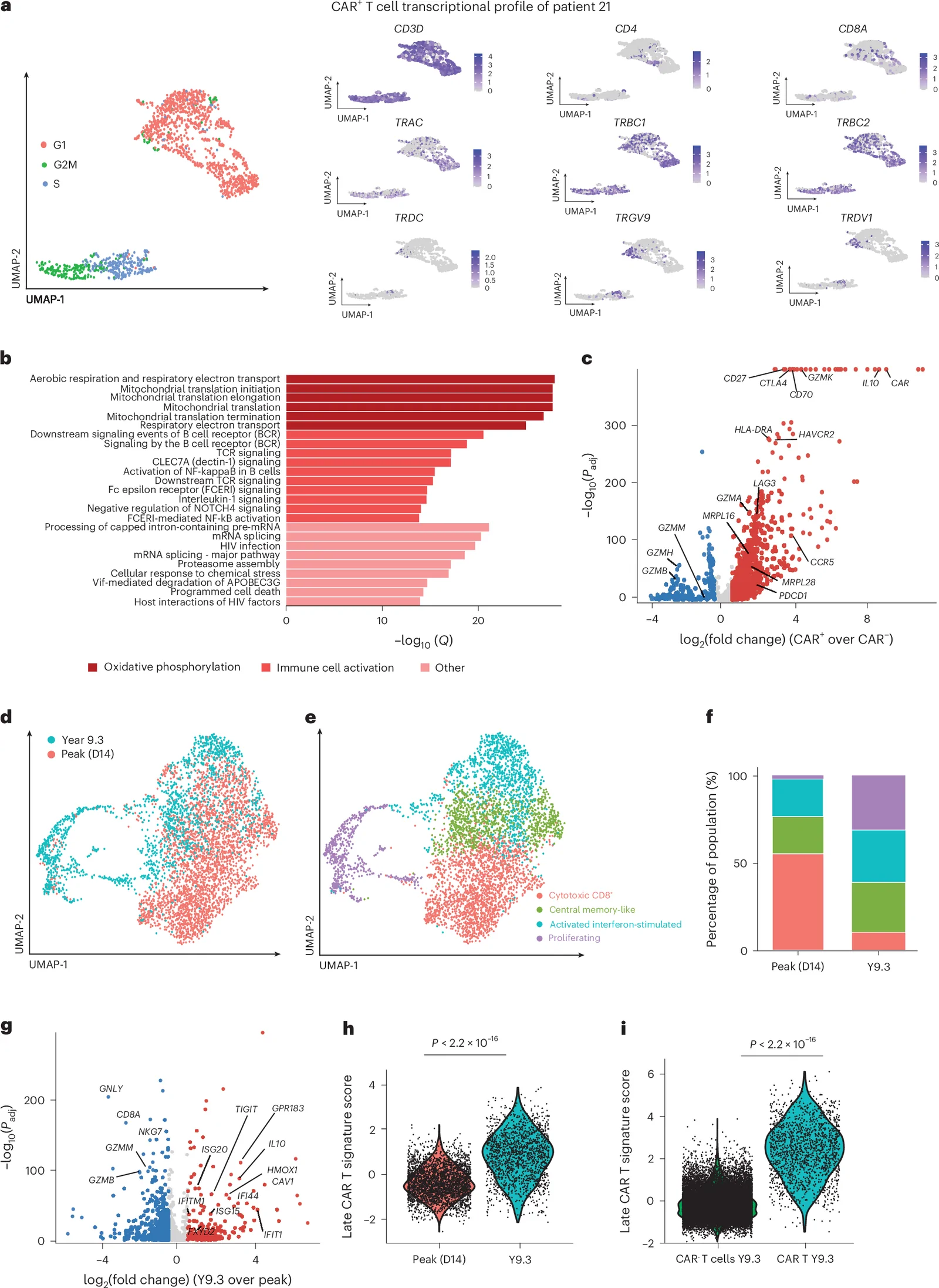
Long-term CAR T cell transcriptional profile. **a**, Uniform manifold approximation and projection (UMAP) of year 9.3 CAR T cells color-coded according to cell-cycle phase. Cell-cycle phase was calculated in the CAR T cell-only object. Feature plots show *CAR*, *CD4*, *CD8* and *TCR* genes. **b**, Reactome pathway enrichment analysis comparing CAR^−^ versus CAR^+^ T cells in G1 phase. Cell-cycle phase was assigned in the combined lymphocyte population. The top 25 pathways are shown. **c**, Differential gene expression analysis of CAR^−^ versus CAR^+^ T cells in the G1 phase. Cell-cycle phase was assigned in the combined lymphocyte population. Blue dots mean higher in CAR^−^, while red dots mean higher in CAR^+^. Differential expression analysis was performed using a two-sided Wilcoxon rank-sum test with Bonferroni correction for multiple testing. **d**, Peak time point and year 9.3 integrated CAR T UMAP color-coded according to sample of origin (red, peak time point; light blue, year 9.3). **e**,**f**, Unbiased clusters of the integrated year 9.3 and peak CAR T (**e**) and relative proportion distribution within each sample; bar plots are color-coded according to the UMAP color cluster. Cytotoxic CD8^+^ CAR T cells are predominant at the peak time point (**f**). **g**, Differential gene expression analysis of CAR T cells in G1 phase year 9.3 versus peak. Blue dots mean higher in peak, while red dots mean higher in year 9.3. Differential expression analysis was performed using a two-sided Wilcoxon rank-sum test with Bonferroni correction for multiple testing. **h**, Comparison of the ‘late’ CAR T signature score, based on the normalized *z*-score expression between the CAR T at the peak and the year 9.3 time point (*P* = 6.70 × 10^−291^). **i**, Comparison of the ‘late’ CAR T signature score, based on the normalized *z*-score expression between the CAR^+^ T at year 9.3 and CAR^−^ T cells at the same time points (*P* < 1 × 10^−291^). The *P* value for the score was calculated using a two-sided Wilcoxon rank-sum test adjusted for multiple comparisons. Significance was set at *P* < 0.05. Source data

**Extended Data Fig. 3:**
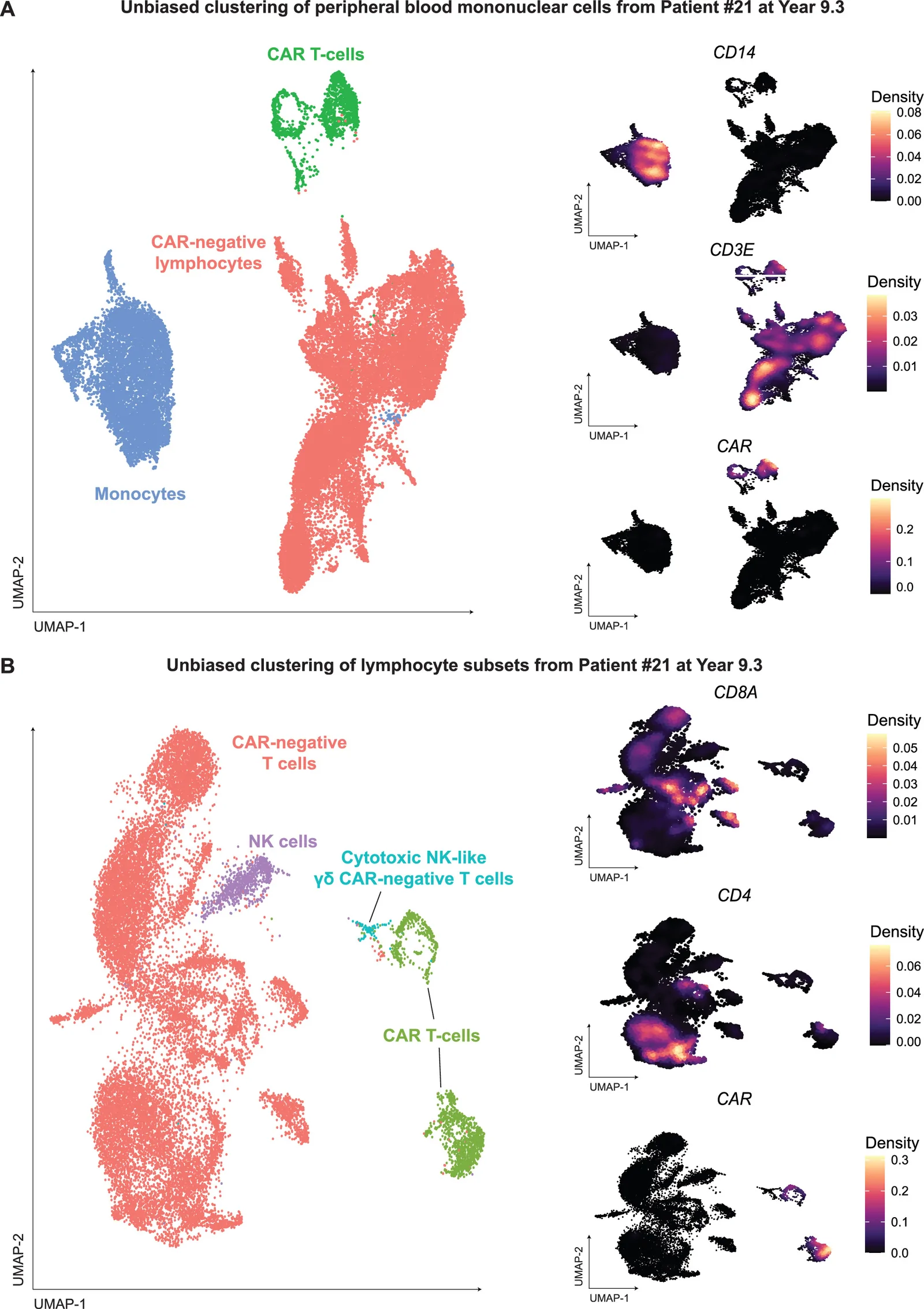
Single cell annotation Patient #21 peripheral blood mononuclear cells year 9.3. (**A**) Uniform Manifold Approximation and Projection (UMAP) of peripheral blood mononuclear cells from patient #21 at year 9.3 post-CART infusion (left). Expression of selected markers used for cluster annotation is shown on the right. (**B**) Uniform Manifold Approximation and Projection (UMAP) of the lymphocytes population from patient #21 at year 9.3 post-CART infusion (left), expression of selected markers used for cluster annotation is shown on the right.

To define the characteristics and functionality of long-lived CAR T cells, we performed single-cell RNA sequencing (scRNA-seq) with paired T cell receptor (TCR) repertoire analysis on peripheral blood mononuclear cells (PBMCs) sorted to enrich for CAR^+^ T cells from patient 21, collected 9.3 years after infusion. This approach yielded 7,267 monocytes, 18,344 CAR^−^ T cells, 836 natural killer (NK) cells and 1,543 CAR^+^ T cells (Extended Data Fig. 3a,b and Supplementary Figs. 6 and 7). Consistent with the flow cytometry results, most CAR^+^ T cells were DN (Fig. 3a). More than half of the CAR^+^ population exhibited an activated, interferon-driven transcriptional program (*CD27*, *CD69*, *IRF1*, *IFI6*, *TIGIT*, *LAG3*) and active proliferation (*MKI67*, *PCNA*), suggesting ongoing long-term activation (Fig. 3a and Supplementary Figs. 8 and 9). We also observed robust expression of cytotoxicity genes (*GZMK*, *PRF1*, *NKG7)*, further confirming the maintenance of a functional cytotoxic program within the CAR^+^ compartment (Supplementary Figs. 8 and 9). Finally, to determine whether the enrichment of DN cells reflected a predominance of γδ CAR T, we analyzed TCR gene expression and found that most CAR T cells exhibited a conventional αβ TCR profile, whereas only a small subset (8%) expressed *TRD* (*TRDV*, *TRDC*) and *TRG* (*TRGC1*, *TRGC2* and *TRGV9*) genes (Fig. 3a).

**Extended Data Fig. 4:**
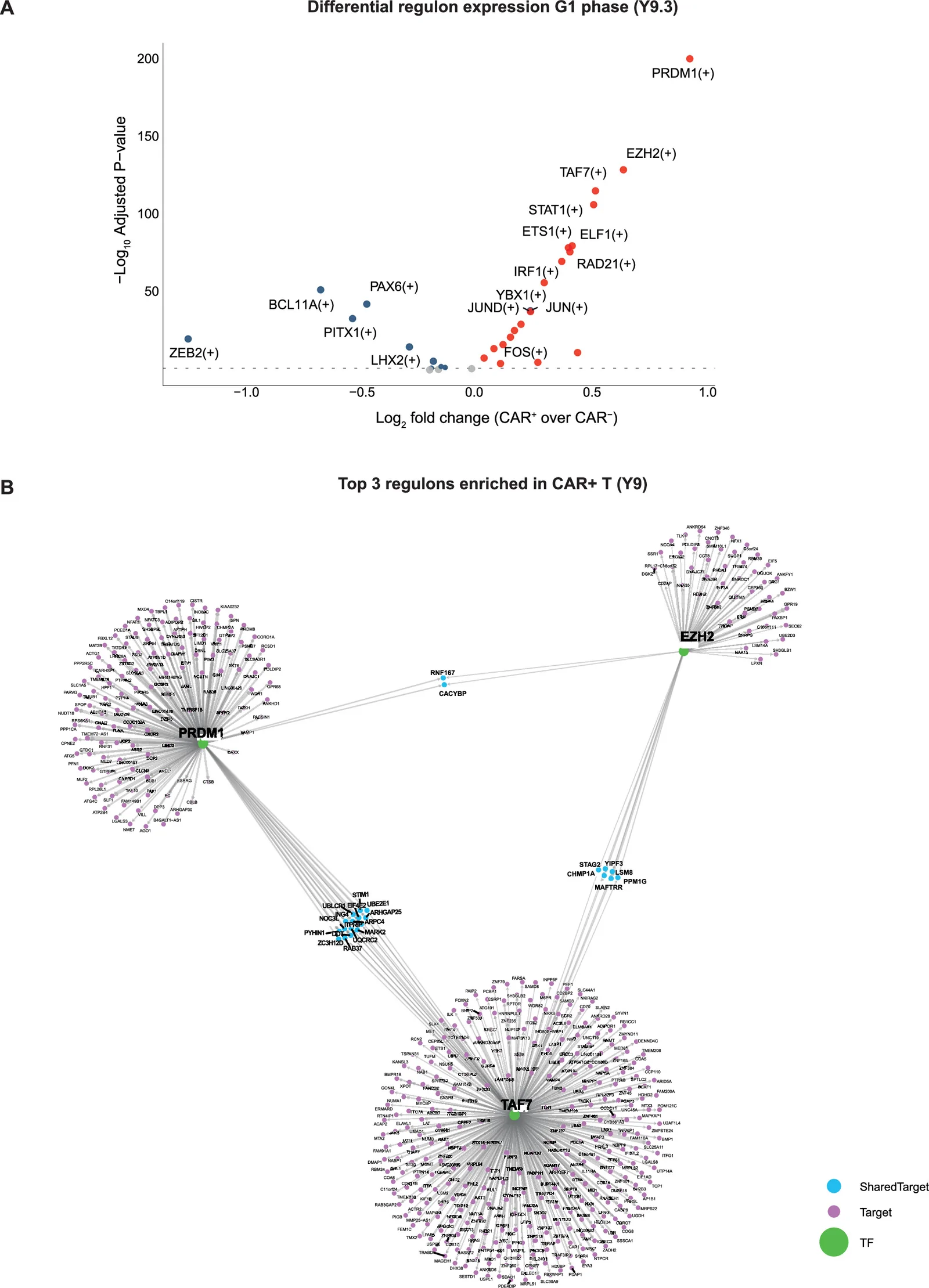
Regulon analysis in CAR+ T compared to CAR−T cells in patient #21 Y9.3. (**A**) Volcano plot showing differential regulon activity in CAR^+^ T cells compared with CAR^−^ T cells at year 9.3. The x-axis represents log_2_ fold change (CAR^+^ vs CAR^−^ T cells), and the y-axis shows −log_10_ adjusted p-values. Regulons significantly enriched in CAR^+^ T cells are highlighted in red, whereas those enriched in CAR^−^ T cells are shown in blue. Selected transcription factors are annotated. Analysis was restricted to G1 phase cells. (**B**) Gene regulatory network of the top three regulons enriched in CAR^+^ T cells at year 9.3. Transcription factors (TFs; green nodes) and their predicted target genes (purple nodes) are shown, with shared targets highlighted in blue. Analysis was restricted to G1 phase cells.

To determine whether the activation and proliferation transcriptional programs were specific to CAR^+^ T cells, we compared cell-cycle status between CAR^+^ and CAR^−^ T cells in the lymphocyte population (Extended Data Fig. 3b and Supplementary Fig. 10a); whereas 66% of CAR^+^ cells were in G2/M or S phase, only 35% of CAR^−^ T cells were proliferating, highlighting sustained CAR^−^-specific proliferation (Supplementary Fig. 10b). We next examined CAR^+^ versus CAR^−^ T cells within the G1 phase. Unbiased pathway enrichment analysis identified that CAR^+^ T cells were enriched for aerobic metabolism and immune cell activation transcriptional programs (Fig. 3b). Differential gene expression analysis identified that CAR^+^ T cells showed higher expression of mitochondrial metabolism (*MRPL16*, *MRPL28*) and activation/exhaustion gene (*HLA-DR*, *CD38*, *PDCD1*, *LAG3*, *HAVCR2*) and produced the regulatory cytokine *IL-10* (Fig. 3c, Supplementary Table 5 and Supplementary Fig. 11). Finally, regulon analysis revealed enrichment of transcriptional programs associated with T cell exhaustion (*PRDM1*), activation (*STAT1*, *TAF7*and *IRF1*) and memory persistence (*JUN* and *FOS*) within the CAR^+^ T cell compartment. In contrast, CAR^−^ T cells were enriched for the *ZEB2* regulon, a transcriptional program associated with effector CD8^+^ T cell responses to infections, which is consistent with the patient’s clinical history (Extended Data Fig. 4 and Supplementary Table 6)^25^.

We next investigated whether the transcriptional features of the long-term-persisting DN CAR^+^ T cells were already detectable at the peak of expansion. To test this, we integrated 3,179 CAR^+^ T cells collected at the expansion peak with those identified at the long-term follow-up (Fig. 3d–g and Supplementary Figs. 12 and 13). Notably, year 9.3 CAR^+^ T cells localized to a UMAP region highly enriched for proliferating and DN CAR^+^ T cells, whereas peak CAR^+^ T cells predominantly mapped to a CD8^+^ cytotoxic cluster characterized by high expression of *GZMB* and *GZMH* (Fig. 3d–g and Supplementary Figs. 12 and 13), indicating a drastically different transcriptional profile. Unbiased pathway enrichment analysis identified interferon signaling as the top enriched pathway in long-term CAR^+^ T cells (Supplementary Figs. 14). Consistently, differential expression analysis revealed a marked upregulation of interferon-related genes (*IFI6*, *IFI44*, *IFITM1* and *ISG15*), together with additional genes previously reported in long-term persisting CAR T cells in B-ALL^17^ (*GPR183*, *HMOX1*, *FXYD2* and *CAV1*) (Fig. 3g and Supplementary Table 7). To further validate these findings, we generated a composite ‘late CAR T signature’ score based on genes defined by Anderson et al.^18^ and observed significantly higher scores in long-term CAR^+^ T cells compared with those at the expansion peak (*P* < 2 × 10^−16^; Fig. 3h)^17^. Moreover, the high ‘late CAR T signature’ observed at year 9.3 was restricted to the CAR^+^ T cell compartment, indicating that this transcriptional program is specific to CAR^+^ T cells rather than a global T cell phenomenon (Fig. 3i).

These findings indicate that in patient 21, long-lived CAR T cells remain metabolically and functionally engaged, progressively transitioning from a cytotoxic CD8^+^ phenotype toward a DN state that adopts a late transcriptional program characterized by an activated effector-memory-like state.

### Validation of the phenotypic and transcriptional profile in long-term-persisting CAR T cells in patients with NHL, B-ALL and CLL

**Fig. 4:**
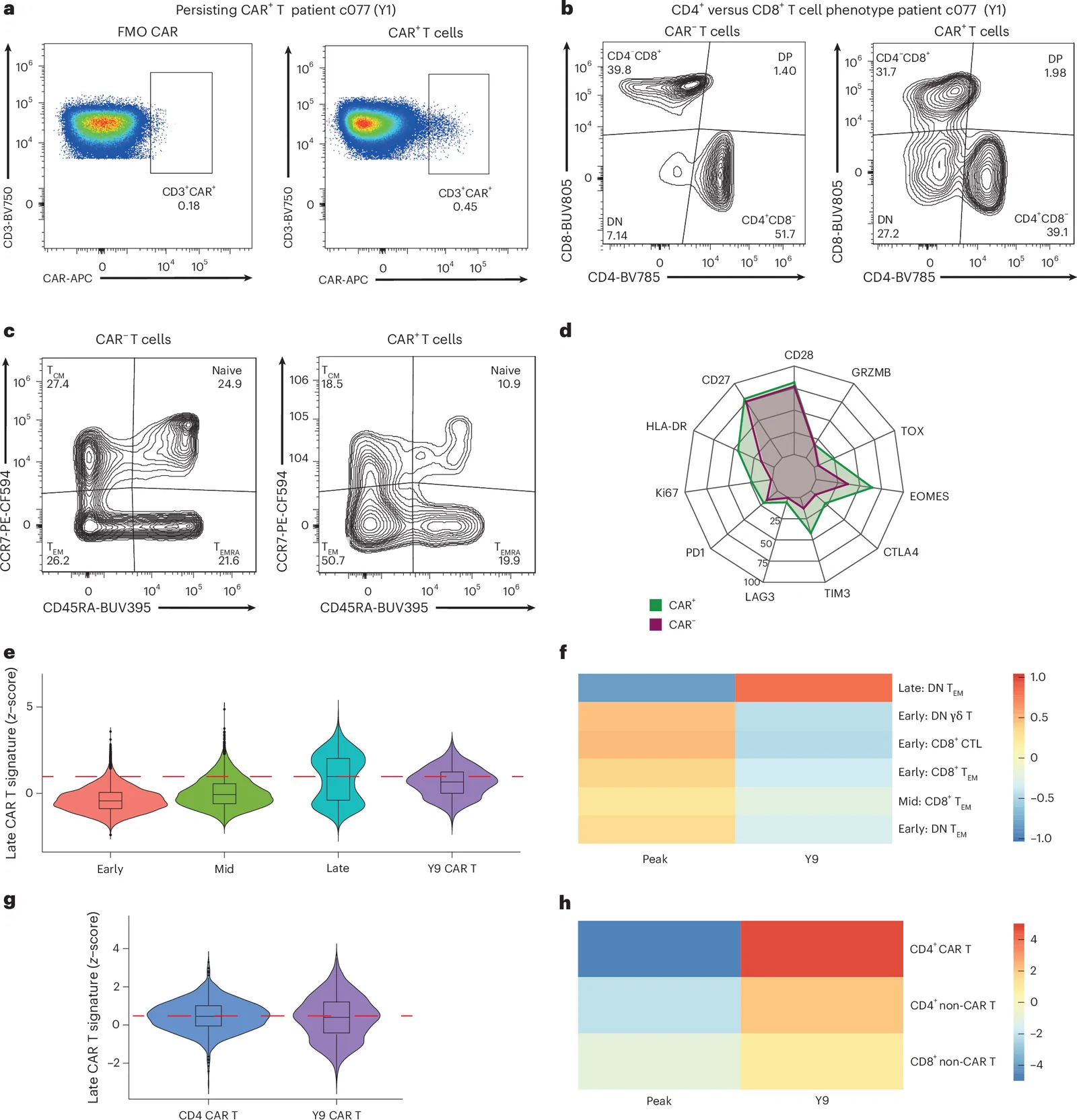
Long-term persisting CAR T cells share phenotypic and transcriptional features across diseases and CAR T products. **a**, Representative gating strategy for CAR^+^ T cells in patient c077 at year 1 (Y1) after tisagenlecleucel infusion. Gating was defined using a CAR fluorescence minus one control. **b**, Distribution of CD4^+^, CD8^+^, DP and DN populations among CAR^+^ T cells compared with CAR^−^ T cells in patient c077. **c**, Memory phenotype distribution of CAR^+^ T cells compared with CAR^−^ T cells in patient c077. Memory subsets were defined as follows: naive (CD45RA^+^/CCR7^+^), T_CM_ (CD45RA^−^/CCR7^+^), T_EM_(CD45RA^−^/CCR7^−^) and T_EMRA_ (CD45RA^+^/CCR7^−^). **d**, Radar plot showing the percentage of cells expressing activation, exhaustion and proliferation markers in CAR^+^ T cells compared with CAR^−^ T cells. **e**, Late signature score across early, mid and late CAR^+^ T cell populations from the dataset by Anderson et al.^18^ (total CAR T cells from the CARPALL dataset: 55,940 cells), compared with year 9.3 (Y9) CAR T cells from patient 21. Analysis was restricted to G1 phase cells. The box plots show the per-cell distribution of the late score as defined by the late-persisting CAR T signature. The dotted line represents the median of the late CAR T from the CARPALL study. The box plots show the first quartile (the lower end of the box), the third quartile (upper end of the box) and the median values (center line) per dataset. The ‘whiskers’ extend from the ends of the box to a maximum and minimum of 1.5 times the interquartile range beyond the box. Outliers are shown as dots. **f**, Heatmap showing logistic regression-based cell-to-cell similarity between early, mid and late CAR T cells from the Anderson et al. dataset and both Y9 and peak CAR^+^ T cells from patient 21. Predicted similarity scores are shown on the logit scale; higher values indicate greater transcriptional similarity to the corresponding reference state. Analysis was restricted to G1 phase cells. **g**, Late signature score in Y9 CAR^+^ T cells from the dataset of Melenhorst et al.^17^ compared with Y9 CAR^+^ T cells from patient 21. Analysis was restricted to G1 phase cells. The box plots show the per-cell distribution of the late score as defined by the late-persisting CAR T signature. The dotted line represents the median of the CD4^+^ persisting CAR^+^ T cells (total: 819 cells). The box plots show the first quartile (the lower end of the box), the third quartile (upper end of the box) and the median values (center line) per dataset. The ‘whiskers’ extend from the ends of the box to a maximum and minimum of 1.5 times the interquartile range beyond the box. Outliers are shown as dots. **h**, Heatmap showing logistic regression-based cell-to-cell similarity between Y9 CAR^+^ T cells from the dataset of Melenhorst et al.^17^ and both Y9 and peak CAR^+^ T cells from patient 21. Predicted similarity scores are shown on the logit scale; higher values indicate greater transcriptional similarity to the corresponding reference state. Analysis was restricted to G1 phase cells. Source data

To assess the generalizability of the findings from patient 21 in NHL, we analyzed eight additional patients with NHL treated with commercial tisagenlecleucel for whom we had available samples at 1–3 years after infusion. CAR^+^ T cells were detectable in one patient using flow cytometry (patient c077, 0.45% of total T cells; Fig. 4a, Supplementary Fig. 15 and Supplementary Table 8). In patient c077, persisting CAR^+^ T cells were enriched for DN T cells compared to their CAR^−^ counterparts (27% versus 8%) and predominantly displayed an effector memory phenotype (51% versus 26%) (Fig. 4b,c). Consistent with what was observed in patient 21, these cells exhibited a proliferative, activated–exhausted phenotype marked by increased HLA-DR, TIM-3 and Ki-67 expression (Fig. 4d), suggesting a similar evolutionary trajectory.

To further validate a shared transcriptomic drift in very-long-persisting CAR T, we evaluated whether the transcriptional program of patient 21 CAR^+^ T cells at year 9.3 overlapped with the profiles described in persisting CAR^+^ T cells in other hematological malignancies, such as B-ALL and CLL. Persisting CAR T cells from patient 21 exhibited a ‘late signature score’ comparable to long-term CAR^+^ T cells reported in pediatric B-ALL by Anderson et al.^18^. Unbiased logistic regression further showed that peak CAR T cells from patient 21 aligned with early and intermediate CAR T cell states, whereas year 9.3 persisting cells shared transcriptional similarity with long-lived CAR T cells observed in acute leukemia (Fig. 4e,f). Similarly, decade-long persisting CAR T cells in CLL (patient 1, year 9.3), as reported by Melenhorst et al.^17^, despite a predominance of CD4^+^ cells, exhibited both a comparable ‘late CAR T signature’ score and a similar global transcriptional profile (Fig. 4g,h).

Collectively, these findings suggest that long-lived CAR T cells converge on a shared trajectory of functional maturation and persistence across disease settings and CAR T cell products.

### Persisting CAR T cells are oligoclonal and are already present at peak expansion

**Fig. 5:**
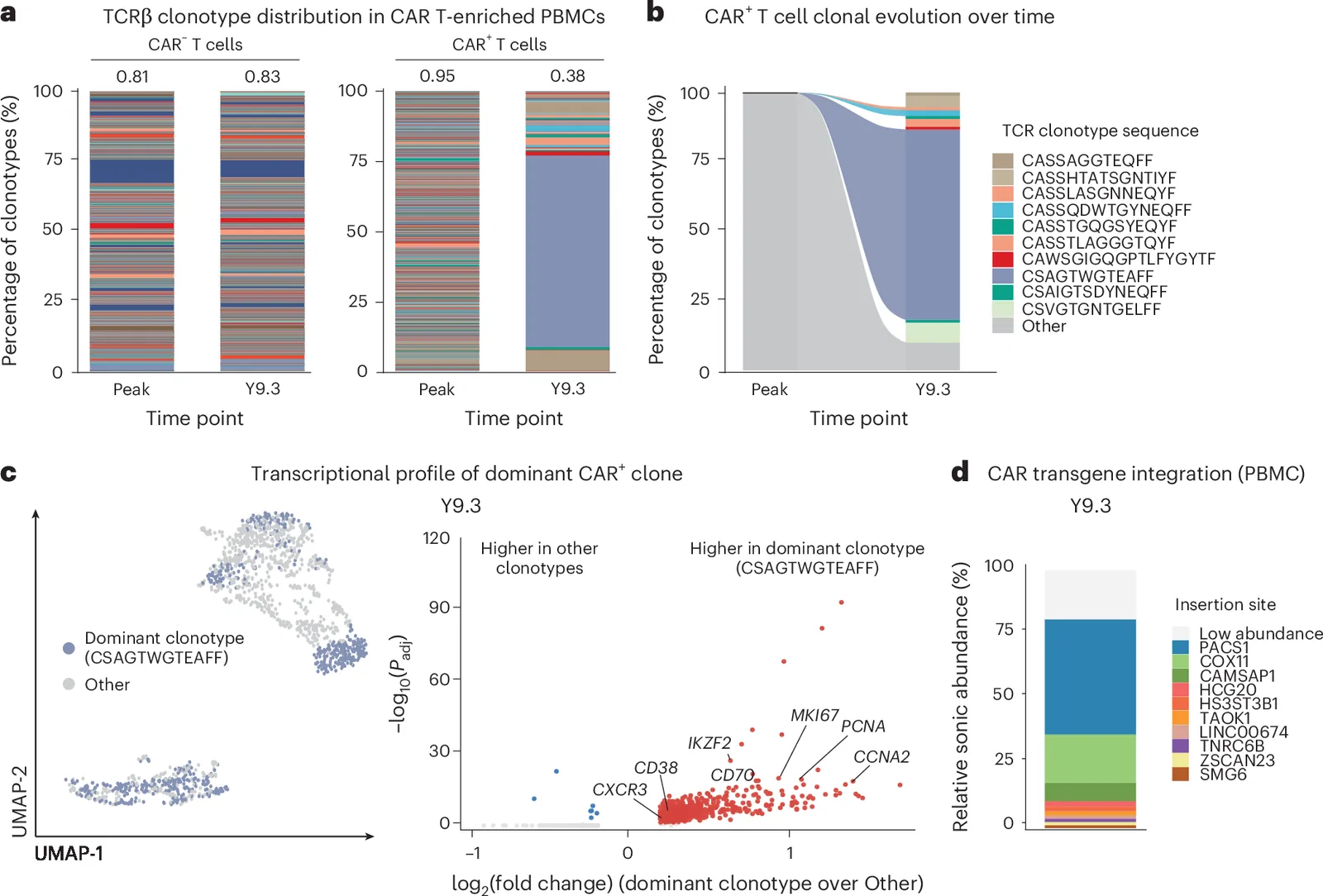
Long-term CAR T cells are oligoclonal. **a**, Stacked bar plot representing the TCR β repertoire in CAR^−^ and CAR^+^ T cells at both peak and year 9.3, showing a marked reduction in normalized Shannon entropy exclusively within the CAR T compartment. As these analyses were performed in a single patient with one repertoire measurement per time point, data are descriptive and no formal statistical comparison was performed. **b**, Alluvial plot tracing the ten most abundant TCRβ clonotypes at year 9.3 back to their representation at peak. Oligoclonal selection was consistent with reduced normalized Shannon entropy. **c**, Projection of the dominant β-chain clonotype on the UMAP of year 9.3 CAR T from patient 21, highlighting its enrichment within cluster-expressing markers of proliferation and activation (left). Right: differential gene expression analysis comparing the dominant clonotype to the other clones within the CAR T population at year 9.3; genes with an absolute log_2_(fold change) greater of 0.25 are plotted. **d**, CAR integration-site analysis at year 9.3 confirms oligoclonality of the CAR^+^ T cell population. Source data

Finally, we analyzed TCR repertoire dynamics to assess whether these phenotypic shifts reflected clonal selection. At peak expansion, we identified 1,593 unique clonotypes within the CAR^+^ compartment and 2,598 within CAR^−^ T cells, whereas at year 9.3 after infusion, 68 clonotypes were detected in CAR^+^ T and 4,239 in the CAR^−^ T cell compartment. Normalized Shannon entropy within CAR^−^ T cells remained stable from peak to year 9.3 (Shannon index 0.81 versus 0.83), while in CAR^+^ T cells entropy declined markedly (0.95 versus 0.38), which is consistent with oligoclonal CAR T cell persistence (Fig. 5a). In line with this observation, a single clonotype (CSAGTWGTEAFF) accounted for nearly 70% of CAR^+^ T cells at year 9.3, whereas it represented less than 0.1% at the peak time point, where it localized to both DN and CD8^+^ clusters (Fig. 5b and Supplementary Fig. 16). The dominant clonotype did not match any known antigen specificities annotated in the VDJ database^26^. Additionally, projection of this clonotype onto the year 9.3 CAR T UMAP revealed localization within proliferating and activated/early exhausted features, supporting a role in sustained immune surveillance (Fig. 5c and Supplementary Figs. 8 and 9). Consistent with this proliferative and activated state, the dominant clonotype at year 9.3 displayed upregulation of proliferation-associated genes (*MKI67*, *PCNA*) and activation or effector-related genes (*CD38*, *CD70*, *CXCR3*) relative to other coexisting clones^27,28^ (Fig. 5c and Supplementary Table 9).

Finally, to exclude the possibility that long-term CAR T persistence resulted from vector integration within oncogenes or from gene-driving T cell proliferation, we performed integration-site analysis on the year 9.3 sample and identified 111 unique insertion sites. Notably, oligoclonality was confirmed at the integration-site level (Gini index = 0.81), with 45% of insertions mapping to the *PACS1*, 19% to *COX11* and 7% to *CAMSAP1*; however, none of the dominant integration sites involved genes previously associated with enhanced CAR T persistence (Fig. 5d)^29^.

Altogether, these results highlight that CART19 can maintain durable CAR T persistence through selective persistence of functionally competent clones rather than insertional mutagenesis. In summary, we identified a long-lived DN αβ T_EM_-like CAR T subset, metabolically oxidative phosphorylation-fit, GZMK^high^ (therefore, nonterminal), checkpoint-high but still proliferative and cytotoxic, capable of intermittent antigen-triggered expansions that sustain remission, a ‘guarded throttle’ program that maintains efficacy while limiting inflammatory toxicity, but at the cost of persistent B cell aplasia and late infectious risk.

## Discussion

In this work, we demonstrate decade-long functional persistence of 4-1BB-costimulated CD19 CAR T cells in adults with B-NHLs. We describe that over time, these long-lived CAR T cells switch to a CD4/CD8 DN activated, effector-memory-like state. We validate our findings in additional patients with NHL and B-cell leukemia treated with anti-CD19 CAR T immunotherapy, recognizing a shared differentiation path for long-term-persisting CAR T across multiple B cell malignancies^17,18^.

Durable CAR T cell persistence and prolonged B cell aplasia correlate with sustained remission in B-ALL^13,30–32^ and also outside hematological malignancies, including neuroblastoma, where persistence was associated with long-term survival^21^. On the other hand, the contribution of long-term persistence in B-NHL was less well defined, with small single-center data supporting that CAR T persistence is associated with prolonged progression-free survival^33^. Notably, in that study, 4-1BB-costimulated products had the longest persistence^33^, which is consistent with the notion that 4-1BB signaling supports mitochondrial fitness, oxidative metabolism and memory-like differentiation, whereas CD28-based CAR T cells are more glycolytic, expand rapidly and show reduced persistence^34–36^. In our trial cohort, CART19 persistence was higher among long-term responders during the first 2 years after infusion^15^. However, the long-term trajectory of CART19 persistence, as well as the phenotype, functional capacity and contribution of persisting CAR T cells to long-term responses, remained unknown. In this decade-long persistence follow-up, CAR T cells remained detectable beyond 5 years in more than 60% of long-term responders, supporting a sustained association between CART19 persistence and long-term remission. Prolonged CAR T cell persistence may be particularly beneficial in indolent diseases, where it could enable ongoing immune surveillance, mitigate late relapses and potentially contribute to prolonged remission. In line with these hypotheses, recent data from the ELARA trial highlighted long-term CAR T persistence in long-term complete responder patients^37^. However, durable remissions also occurred in patients without long-term persisting CAR T cells, indicating that it is not an absolute requirement and that factors such as tumor burden, microenvironment depth of initial response and early CAR T kinetics may suffice for durable disease control in some cases. Long-term CAR T cell persistence can contribute to the observed sustained B cell aplasia, thereby increasing the risk of infections and nonrelapse mortality, raising questions about the net clinical benefit of prolonged CAR T persistence. While the role of persisting CAR T cells in immune surveillance and prevention of lymphoma relapse remains under investigation, persistence is generally favored when infectious complications can be effectively managed with standard outpatient strategies, including IVIG replacement and appropriate antimicrobial therapy. However, in the setting of prolonged B cell aplasia associated with severe or recurrent infections, there is a clear rationale to limit long-term CAR T cell persistence. Potential strategies to address this challenge include the development of anti-CAR ‘antidote’ approaches^38^ to selectively deplete CAR^+^ T cells, as well as innovative therapies designed to target malignant B cells^39^ while sparing normal B cell compartments.

In our study, we show that, in a significant subset of patients with LBCL and FL, 4-1BB-costimulated CART19 can be detected up to 10 years after initial infusion. Persisting CAR T cells remained phenotypically detectable in patient 21 at year 9.3 after infusion. In this patient, CAR T cells gradually shifted during the first 2 years after infusion toward a predominant CD4/CD8 DN αβ phenotype. Transcriptomic analysis revealed features consistent with prior reports in CLL and B-ALL, including cytotoxic markers (*GZMK*, *PRF1*, *NKG7*) and activated–exhausted cell state, with enrichment of *FXYD2*, *HMOX1* and *GPR183* expression^17,18^. Persisting CAR T cells displayed higher expression of *GZMK* compared to CAR^−^ T cells, whereas *GZMB*, a canonical marker of cytotoxicity, was reduced. *GZMK*^+^ subsets are commonly less terminal, more migratory/patrolling, still capable of killing (*PRF1*, *NKG7* present) and producing inflammatory mediators. This might explain durable control without overt toxicity and the ability to expand on demand^40–42^. Cytotoxic tumor-infiltrating lymphocytes expressing *GZMK* have been described in bladder cancer, where they contribute to tumor clearance, suggesting a role for antineoplastic immune surveillance of the persisting CAR T cells^43^. Notably, our understanding of the role of *GZMK*^+^ T cells is rapidly expanding, with recent studies identifying these populations as pivotal mediators in several chronic immune-mediated conditions, including rheumatoid arthritis, post-allogeneic stem cell transplantation chronic graft-versus-host disease-associated bronchiolitis obliterans syndrome, chronic rhinosinusitis and asthma, through activation of pro-inflammatory cascades^43–47^. Together, these findings raise the possibility that GZMK^+^ CAR T cells represent a specialized, inflammation-competent cytotoxic subset that may mediate sustained immune vigilance during long-term remission. Persisting CAR T cells have high expression of *FXYD2*, *HMOX1* and *GPR183*. The role of *FXYD2*, the γ-subunit of Na^+^/K^+^-ATPase, in T cells has not yet been explored, whereas *HMOX1*, involved in iron metabolism, represents a crucial factor for T cell mitochondrial function and cellular metabolism, factors that have emerged as key determinants of long-term clinical response and CAR T persistence^48^. Finally, *GPR183*, a chemotactic G-protein-coupled receptor, is implicated in immune cell positioning within secondary lymphoid tissues, suggesting a potential role in long-term immune surveillance^49^. The specific functional role of these transcriptomic ‘stigmata’ will require future mechanistic studies.

Long-term-persisting CAR T cells have been previously characterized in CLL (10 years, two patients) and pediatric B-ALL (5 years, seven patients), but not in NHL. CD4^+^ CAR T cells dominated the persisting population in CLL^17^, while in B-ALL, persisting CAR T cells exhibited a DN phenotype^18^. Despite these phenotypic differences, both studies converged on a similar finding: persisting CAR T cells evolved toward an effector-like memory state and acquired a distinctive transcriptional profile, with expression of a ‘late signature’ characterized by genes such as *FXYD2*, *HMOX1* and *GPR183*. In our cohort, logistic regression revealed that patient 21’s year 9 CAR T cells exhibited global transcriptional similarity to long-term persisting CAR T cells in B-ALL and CLL, confirming that there is a conserved trajectory of transcriptomic evolution across hematological malignancies and CAR T products.

In our study, we also analyzed the clonality of CAR T cells. We observed a reduction in clonotype-normalized entropy in CAR^+^ T cells compared with CAR^−^ T cells. This mirrors previous findings, which demonstrated marked oligoclonal dominance in patients achieving decade-long remission after CART19 for CLL^17^. In contrast, long-term analysis of the CARPALL trial^18^ showed sustained polyclonality among persisting CAR T cells, suggesting that clonality trajectories may be dictated by product-intrinsic properties and manufacturing biology^18^. The integration sites identified in patient 21 have not been previously associated with CAR T cell persistence^29^. While the impact of *COX1* and *CAMSAP1* integration sites has not been previously characterized, *PACS1* is a relatively common lentiviral integration site predominantly detected in the manufactured CAR T infusion product, rather than post-infusion samples, indicating that the dominant *PACS1* integration observed in this study probably reflects vector insertion bias rather than a mechanistic driver of long-term CAR T persistence^29,50^.

While analyzing long-term CAR T cell persistence, we observed intermittent flares and contractions of CAR transgene levels over time. Potential explanations include technical variability in assay sensitivity across years, as well as cycles of CAR T activation and quiescence driven by transient B cell reconstitution, subclinical tumor or tumor antigen exposure or conditions resulting in reactive lymphocytosis, such as virosis. This dynamic pattern is consistent with the model proposed in CLL^16^, in which persisting CAR T cells periodically re-engage with antigen to sustain immune surveillance^17^, and parallels the re-expansion of Epstein–Barr virus-specific T cells observed during Epstein–Barr virus reactivation^51^. In this context of recurrent or chronic antigen stimulation, phenotypic adaptation of persisting CAR T cells may also occur. Supporting this, CAR T cells from patient nos. 21 and c077 were enriched for DN T cells, which is consistent with prior reports showing that DN T cells can arise through progressive CD8 downregulation in response to chronic antigen exposure^18,52^.

Our study has several limitations. First, the modest sample size and incomplete availability of longitudinal biospecimens limited our ability to comprehensively evaluate the biological determinants of long-term CAR T cell persistence. In particular, detailed phenotypic, transcriptional, clonal and integration-site analyses were feasible in only a subset of patients and were largely driven by observations from a single individual with exceptionally prolonged persistence. Second, although we identified phenotypic and transcriptional features shared with long-term persisting CAR T cells described in B-ALL and CLL, these findings should be considered exploratory and hypothesis-generating. The mechanisms underlying the emergence, maintenance and intermittent expansion of long-lived CAR T cell populations remain incompletely understood. Third, while persistent CAR T cells were associated with sustained B cell aplasia and durable remissions in a subset of patients, our study was not designed to determine whether decade-long persistence directly contributes to disease control or instead reflects favorable host, disease or product characteristics. Future prospective studies with systematic longitudinal sampling and larger patient cohorts will be required to define the determinants of long-term persistence and to establish the contribution of persistent CAR T cells to long-term immune surveillance and durable remission. Despite these limitations, our findings provide a comprehensive characterization of CAR T cell biology after a decade of follow-up in lymphoma and establish a framework for future investigations into the mechanisms and clinical consequences of long-term cellular persistence.

## Methods

### Study design and oversight

The long-term analysis study was conducted in accordance with the Declaration of Helsinki and approved by the University of Pennsylvania Institutional Review Board (IRB). Written informed consent was obtained for review of medical records and for research use of biospecimens. In this study, we report long-term persistence of 38 B-NHL patients who received 4-1BB-costimulated, lentivirally transduced CART19 T cells in the trial (NCT02030834). Patient characteristics have been reported previously^5^.

### Correlative studies

#### CART19 qPCR

For long-term follow-up, CAR T cell persistence testing was performed in the Translational and Correlative Studies Laboratory at the University of Pennsylvania, using established standard operating procedures. PBMC-derived DNA was isolated using the automated Qiacube Connect instrument method (QIAGEN). A blood sample and all necessary reagents were placed in the instrument. DNA released after sample lysis and proteinase K digestion was dehydrated and bound to silica particles, built into the separation column. Centrifugation was used to wash out all other unbound components before the captured rehydrated DNA was eluted. The DNA was frozen and kept at −80 °C until later use. Quantification of transgene in peripheral blood-derived DNA was performed using real-time qPCR with reverse transcription (RT–qPCR), as described previously^1,5,53^. Briefly, RT–qPCR analysis was performed using an assay amplifying and detecting a specific integrated CAR transgene sequence. Specificity was ensured at two levels: (1) via a set of specific forward and reverse primers and (2) via a specific fluorescent TaqMan technology probe (Applied Biosystems). Testing precision and sensitivity was achieved using triplicates of 200 ng of genomic DNA (gDNA) for standards and patient samples per time point (or maximum available). The accuracy of transgene copy detection was achieved by referring the tested sample against a standard curve created based on the values obtained from nine standards covering a range of five up to 1 × 10^6^ copies of lentivirus plasmid spiked into 200 ng of nontransduced control human gDNA. The number of copies of plasmid present in the standard curve was verified using digital PCR with the same primer/probe set and performed on a QIAcuity One (QIAGEN).To control for the quantity of interrogated DNA, we performed a parallel amplification reaction using 10 ng of gDNA and a primer/probe combination specific for a nontranscribed genomic sequence upstream of the *CDKN1A* (p21) gene. These amplification reactions generated a correction factor to adjust for calculated versus actual DNA input. Combining the transgene copy number and the actual amount of tested DNA enabled the determination of copy number per unit of DNA. Final copies of transgene per μg of DNA were calculated according to the following formula: copies per μg of gDNA = (copies calculated from CAR T standard curve) × correction factor/(amount of DNA evaluated in ng) × 1,000 ng.

Previously performed CAR19 qPCR data were acquired as described previously^1,5,53^. Limit of detection for CAR19 qPCR was 25 copies per µg of DNA from CAR T cell infusion through 24 months; 50 copies per µg of DNA from previously analyzed samples collected from month 24 to year 7; and five copies per µg of DNA for newly performed assays between year 7 and 10 (long-term phase). Differences between LBCL and FL were assessed using the nonparametric Wilcoxon rank-sum test, with statistical significance defined as a two-sided *P* < 0.05. Differences in expansion based on lymphodepletion regimens (Cy-based versus bendamustine, carboplatin-based regimen (*n* = 1) was excluded from the comparison) were assessed using the nonparametric Wilcoxon rank-sum test with correction for multiple testing, with statistical significance defined as a two-sided *P* < 0.05.

#### Multiparameter flow cytometry analysis of CAR19 expression and cellular phenotypes

Multiparametric flow cytometry was performed on all available peripheral blood samples with molecularly or phenotypically detected CART19. The CAR19 was detected intracellularly using the MDA CAR19 APC antibody (clone 136.20.1), which is anti-ID CD19scFv protein. This antibody was obtained from the Cooper lab (MD Anderson Cancer Center) and conjugated in-house with the Lightning-Link APC Antibody Labeling Kit (Abcam). Marker expression was evaluated using multicolor flow cytometry.

Frozen PBMCs were thawed and counted. Dead cells were excluded using the LIVE/DEAD Fixable Blue Dead Cell Kit (Thermo Fisher Scientific) for 20 min at room temperature. Further blocking was performed using Human TruStain FcX (BioLegend) for 10 min at room temperature before staining with specific surface marker antibodies in flow buffer (PBS with 1% FCS, 0.02% sodium azide), BD Horizon Brilliant Stain Buffer Plus (BD Biosciences) and True-Stain Monocyte Blocker (BioLegend) for 20 min at room temperature. Finally, cells were washed, fixed and permeabilized using the FOXP3 buffer set (Invitrogen), and stained with intracellular antibodies in permeabilization buffer and BD Horizon Brilliant Stain Buffer Plus for 20 min at room temperature. Then, cells were acquired using a Cytek Aurora flow cytometer equipped with an ultraviolet laser (355 nm), a violet (405 nm), blue (488 nm), green (560 nm) and a red (640 nm) laser, ensuring proper spectral unmixing and compensation. Compensation values (or reference controls) were established using paired normal donor PBMCs, activated PBMCs and cells with CAR19 expression. Data were analyzed using FlowJo. Surface staining antibodies were: anti-CD45RA BUV395 (clone HI100); anti-CD16 BUV496 (clone 3G8); anti-TIM3 BUV615 (clone 7D3); anti-CD28 BUV737 (clone CD28.2); anti-CD8 BUV805 (clone SK1); anti-CD19 BV480 (HIB19); anti-CD3 BV750 (clone SK7); anti-CD95 BB700 (clone DX2); anti-CCR7 PE-CF594 (clone 150503) (all from BD Biosciences); and anti-PD-1 BV421 (clone EH12.2H7); anti-CD27 BV510 (clone O323); anti-CD33 BV570 (clone WM53); anti-CD56 BV605 (clone HCD56); anti-HLA-DR BV711 (clone L243); anti-CD4 BV785 (clone OKT4); anti-CD34 AF647 (clone 581); and anti-CD45 APC-Fire810 (clone HI30) (all from BioLegend); and anti-CD123 SB436 (clone 6H6) and anti-LAG3 PE-Cy7 (clone 3DS223H) (both from Invitrogen). Intracellular staining antibodies were: MDA CAR19 APC (clone 136.20.1); anti-CTLA-4 PE-Cy5 (clone BNI3); and anti-Ki67 AF700 (clone B56) (from BD Biosciences); anti-Granzyme B APC-Fire750 (clone QA16A02) (from BioLegend); anti-EOMES FITC (clone WD1928) (Thermo Fisher Scientific); and anti-TOX PE (clone REA473) (Miltenyi Biotec). Supplementary Table 10 describes the reagents used for this multiparameter flow cytometry. Analysis was performed using FlowJo v10.10 (FlowJo LLC). For high-dimensional analysis, cells were gated on viable, singlet lymphocytes (CD45^+^, CD3^+^, CD14^−^). Unbiased T cell clustering using non-CAR parameters was performed using the FlowSOM plug-in in FlowJo and visualized using *t*-SNE. The following markers were used for FlowSOM annotation: CAR19, GZMB, Ki-67, CD95, CD45, CD45RA, TIM3, CD28, CD8, PD1, CD27, CD56, CD16, HLA-DR, CD4, EOMES, TOX, CCR7, CTLA4, LAG3, CD33.

Previously performed flow cytometry of apheresis, infusion product and post-infusion time points were retrospectively reanalyzed. Data for this analysis were acquired as described previously^1^^,53^.

For the correlation analyses of B cells at apheresis, infusion product characteristics and post-infusion time points with clinical outcomes, long-term response was defined as progression-free survival exceeding 5.4 years, corresponding to the time of the last observed relapse in the cohort^15^.

#### Flow cytometry screening analysis of CAR19 in patients receiving commercial CART19

This analysis was conducted in accordance with the 2024 Declaration of Helsinki and was approved by the IRB at the University of Pennsylvania. Written informed consent for the use of clinical samples was obtained from all patients. PBMC samples were analyzed in adult patients with BCL enrolled in the UPCC37418. Frozen PBMCs were thawed and counted. Cell viability was established using the ViaKrome 808 Fixable Viability Dye (cat. no. C36628, Beckman Coulter) or LIVE/DEAD Blue (cat. no. L34962, Invitrogen). Cells were resuspended in fluorescence-activated cell sorting staining buffer (PBS + 2% FCS) using the following antibodies: anti-CD3 BUV395 (clone UCHT1, cat. no. 563546, BD Biosciences) or anti-CD3 BV750 (clone SK7, cat. no. 747058, BD Biosciences), anti-CD14 Pacific Blue 525 (clone RMO52, cat. no. B00846, Beckman Coulter) or BUV563 (clone M5E2, cat. no. 741360, BD Biosciences), anti-CAR (anti-FMC63-PE (Novartis) or APC (Cooper lab))^5,53^. Data were acquired on a CytoFlex LX Flow Cytometer (Beckman Coulter) or Cytek Aurora flow cytometer. All data analysis was performed using FlowJo v.10.10 (FlowJo LLC). For patient c077, multiparameter flow cytometry was performed using the same methods and panels described in ‘Multiparameter flow cytometry analysis of muCAR19 expression and cellular phenotypes’.

#### Proteomics analysis

Previously generated Luminex data were retrospectively reanalyzed according to the lymphodepletion regimen used (Cy-based versus bendamustine). Data acquisition was performed as described previously^1^^,53^. Measurements below or above the assay limits of detection were excluded; assays with more than 50% nonquantifiable values were not included in the comparison.

#### Single-cell transcriptome profiling and TCR Vβ V(D)J sequencing using 5′ single-cell sequencing

Frozen PBMCs from the peak time point and year 9.3 were thawed and counted. Thawed samples were fluorescence-activated cell sorting-sorted enriching for CAR T (Supplementary Fig. 6 shows the gating strategy) using the following antibodies: LIVE/DEAD aqua (Invitrogen); CD3 (clone UCHT1, BUV395, BD Biosciences), CD45 (clone KC56, FITC Beckman Coulter), CAR (clone NOV1220, PE, Novartis). After sorting, 29,000 single-suspension cells were partitioned using the 10x Genomics Chromium iX single-cell platform (10x Genomics) for an expected capture of 20,000 single cells per sample. A total of two lanes were loaded, each containing two different samples. Single-cell droplets were generated using the Chromium GEM-X single-cell 5′ kit v3 (10x Genomics). Complementary DNA (cDNA) synthesis and amplification, TCR amplification, library preparation and indexing were done using the 10x Genomics human TCR amplification kit and Gene Expression Library Preparation kit (10x Genomics), according to the manufacturer’s instructions. The following PCR cycles were used at each step: 11 PCR cycles for cDNA amplification; 12 and ten cycles for PCR1 and PCR2, respectively, for TCR amplification; six PCR cycles for index incorporation into the final TCR library; and 12 PCR cycles for index incorporation into the final gene expression library. The overall library size was assessed using the 4200 TapeStation and the High-sensitivity DNA 5000 ScreenTape (Agilent Technologies). Concentration was determined using the Qubit Fluorometer 2.0 (Thermo Fisher Scientific). Two gene expression libraries and 2T cell VDJ libraries were pooled according to the reads per cell requirement suggested by 10x Genomics. Next-generation sequencing on the pool was done on the NextSeq 2000 (Illumina) using a P3 100 cycle kit (Illumina), yielding 1.2B total reads, paired-end run with the following run parameters: 28 base pair × 10 base pair (index) × 90 base pair. Reads were aligned to the human reference genome (GRCh38), augmented with a custom reference to detect the CART19 transgene, using Cell Ranger v.8.0.1. TCR sequences were identified using Cell Ranger VDJ and clonotypes were defined based on TCR Vβ CDR sequences. Quality control and downstream analyses were performed in Seurat v.5. Cells with fewer than 200 or more than 7,500 detected genes, or more than 10% mitochondrial gene content, were excluded. TCR processing and clonotype definition were performed using scRepertoire v.2.5.1. Clonotype metadata were integrated into the Seurat object with combineExpression. Seurat objects were converted to SingleCellExperiment v.1.30.1 and processed with scDblFinder v.1.22.0. Cells classified as ‘doublet’ were removed; only ‘singlet’ cells were retained for the downstream analyses. Cell-cycle scoring was performed in Seurat using the updated 2019 gene sets (cc.genes.updated.2019) for the S and G2/M phases. For direct comparisons between CAR^+^ and CAR^−^ T cells, cell-cycle scores and phase assignments were generated in the combined lymphocyte population, which included CAR^+^ T, CAR^−^ T and NK cells. For year 9 CAR T-only reclustering and visualization, cell-cycle scoring was recalculated within the CAR^+^ subset but was not used for direct CAR^+^ versus CAR^−^ T cell comparisons. For longitudinal comparisons between peak and year 9.3 CAR^+^ T cells, cell-cycle scoring was performed in the combined CAR^+^ object containing both time points. Dimensionality reduction was performed via UMAP on the top 15 principal components, using 15 neighbors and two output dimensions. Clustering was performed with FindNeighbors and FindClusters; differential gene expression was analyzed with the Wilcoxon rank-sum test and Bonferroni correction using FindMarkers. Clusters were annotated based on Szabo et al.^54^. CAR T annotation was performed at the cluster level and used to compare CAR^+^ and CAR^−^ T cells. Cells were classified as γδ T cells if they expressed at least one δ-chain gene (*TRDC* or *TRDV1* > 1) and at least one γ-chain gene (*TRGC1*, *TRGC2* or TRGV9 > 1). CAR^+^ clusters from the peak time point and year 9.3 were retained and integrated, accounting for batch effect using Harmony v.1.2.3, specifying the sample of origin as the grouping variable. Differential expression analysis on single-cell data was performed using the Wilcoxon rank-sum test with Bonferroni multiple testing correction. Gene expression densities were visualized with Nebulosa v.1.18.0. Functional enrichment analyses were performed using clusterProfiler v.4.16.0. Gene sets were obtained from ReactomePA (ReactomePA v.1.52.0). The late CAR T signature score was calculated by inputting the ‘late CAR T signature’ gene list from Anderson et al.^18^ (*FXYD2*, *HMOX1*, *GPR183*, *TIGIT*, *HLA-DRA*, *TMEM155*, *FMCR*, *MTSS1*, *TTN*, *DENND2D*, *NUCB2*, *EOMES*, *CAV1*, *LYAR*, *ANTXR2*, *PASK*, *CEP128*, *CMTM7*, *ABHD17B*, *LIMD2*, *GNA12)*. Gene signature scores were calculated using the AddModuleScore function in Seurat; the resulting values were normalized using the scale() function to derive *z*-scores. Clonotype diversity was evaluated using normalized Shannon entropy (adjusted for sample size) across CAR^−^ and CAR^+^ compartments (entropy package v.1.3.2). Clonotype dynamics were visualized as stacked bar plots of proportional abundance and alluvial plots depicting peak-to-year-9 evolution. Only productive TCR rearrangements were used in the assessment of TCR clonotype frequencies. The dominant clonotype was compared against the VDJ reference database^26^.

#### Regulon analysis

A pySCENIC-based AUCell workflow was used to quantify regulon activity across comparison groups. Analyses were performed in the scenic conda environment using pySCENIC (v.0.12.1), pandas (v.2.3.3), NumPy (v.1.23.5), SciPy (v.1.15.2) and matplotlib (v.3.10.8). The input expression matrix was reformatted from gene-by-cell to cell-by-gene format, duplicated gene symbols were merged by summing expression values, and regulon activity scores were calculated using pyscenic aucell with a shared regulon definition file. Only positive regulons (+) were included for downstream analysis. To minimize confounding effects related to proliferation, analyses were restricted to cells in the G1 phase. For each regulon, mean AUCell scores were calculated for each group; the effect size was defined as the log_2_(fold change) of mean regulon activity between the two comparison groups. Statistical significance was assessed using the Wilcoxon rank-sum test, followed by Benjamini–Hochberg correction for multiple testing. Labeled regulons were selected by ranking all tested regulons according to Benjamini–Hochberg-adjusted *P* value and annotating the top 20 most significant regulons.

For regulon network visualization, target genes and their associated weights were parsed from the TargetGenes field to generate a transcription factor-target edge list; shared targets were defined as genes connected to at least two transcription factors. To simplify the network, target genes were ranked within each transcription factor according to regulon weight; the top 15 targets were retained; shared targets were then added back before duplicate transcription factor-target edges were removed. Nodes were classified as transcription factors, unique targets or shared targets; the directed network was visualized with a force-directed layout. Edge width was scaled according to regulon weight; node color indicated node class.

#### Multinomial logistic regression

Multinomial logistic regression was performed using Matrix (v.1.7.4), glmnet (v.4.1-10), dplyr (v.1.1.4), pheatmap (v.1.0.12), AnnotationDbi (v.1.72.0) and org.Hs.eg.db (v.3.22.0). RNA expression data were first prepared to ensure the availability of a normalized data layer, with layer joining applied when required for layered Seurat objects. Genes were selected in a data-driven manner by first identifying genes shared between the reference and query expression matrices, then excluding mitochondrial genes (MT), ribosomal genes (RPL/RPS), TCR genes (*TRAV*, *TRAJ*, *TRBV*, *TRBJ*, *TRBC*, *TRAC*) and immunoglobulin genes (*IGH*, *IGK*, *IGL*). Highly variable genes were identified in the reference dataset using Seurat’s VST-based feature selection (nfeatures = 3,000); the final model features were defined as the intersection between the filtered shared genes and the reference highly variable genes. A multinomial logistic regression model with ridge regularization (alpha = 0) was trained using fivefold cross-validation; class probabilities were generated for each query cell. Predicted probabilities were then averaged across predefined query cell groups, transformed to logit values using a pseudocount (1 × 10^−6^), and visualized as unclustered heatmaps.

#### Integration-site analysis

Integration-site isolation and sequencing was carried out using ligation-mediated PCR as described previously^29^. Briefly, genomic DNA (1,300 ng) was sheared using a Covaris M220 ultrasonicator to achieve a fragment size of 600–900 bp, followed by bead purification and linker ligation. Nested PCR was performed to amplify from the linker to the vector long terminal repeat. Amplified samples were pooled, purified, quantified and sequenced on an Illumina MiniSeq sequencer with a library loading concentration of 1.4 pM with a 20% PhiX spike-in. Sequence reads were quality-controlled, aligned to the human reference genome hg38 and analyzed as described previously^26^.

All integration-site analysis data are available at the NCBI Sequence Read Archive (SRA) under accession no. PRJNA1357863.

Integration-site analysis was carried out at the Viral Molecular High-Density Sequencing Core at the University of Pennsylvania (Research Resource Identifier: SCR_022433).

### Statistics and reproducibility

We analyzed all 38 consecutive patients (24 male and 14 female) infused with CART19 and evaluable for efficacy in the phase IIa study (NCT02030834). All available samples were included in the analysis; therefore, biological sex was not considered in the design of the study and no data were excluded. Statistical analyses are described in the Methods and figure legends. All tests were two-sided unless otherwise specified. Differential gene expression analysis of scRNA-seq data was performed using the Wilcoxon rank-sum test with Bonferroni correction for multiple comparisons. No statistical method was used to predetermine the sample size for this long-term follow-up analysis. Experiments were not blinded; however, analyses were conducted using automated computational pipelines, minimizing subjective bias. Continuous variables are presented as the median and interquartile range and were compared using the Wilcoxon rank-sum test, with correction for multiple testing when appropriate. Categorical variables were compared using the chi-squared test or Fisher’s exact test, as appropriate. Statistical significance was defined as a two-sided *P* < 0.05. All analyses were performed using R v.4.3 and Prism v.10.6.1 (GraphPad Software).

### Reporting summary

Further information on research design is available in the Nature Portfolio Reporting Summary linked to this article.

## Online content

Any methods, additional references, Nature Portfolio reporting summaries, source data, extended data, supplementary information, acknowledgements, peer review information; details of author contributions and competing interests; and statements of data and code availability are available at https://doi.org/10.1038/s41591-026-04578-1.

## Supplementary information

Supplementary Information Supplementary Figs. 1–16.

Reporting Summary

Supplementary Table Supplementary Tables 1–10.

## Source data

Source Data Fig. 2 Data source for Fig. 2.

Source Data Fig. 3 Data source for Fig. 3.

Source Data Fig. 4 Data source for Fig. 4.

Source Data Fig. 5 Data source for Fig. 5.

## Data Availability

All requests for raw and analyzed data and materials are reviewed within 8 weeks by the University of Pennsylvania and the corresponding authors to determine whether they are subject to intellectual property or confidentiality obligations. Patient-related data not included in the paper may be subject to patient confidentiality. De-identified clinical data supporting the findings of this study are available from the corresponding authors upon reasonable request, subject to IRB approval, patient consent restrictions and applicable institutional and federal regulations governing the sharing of human participant data. Any data and materials that can be shared will be released via a material transfer agreement. The integration-site sequencing data have been deposited in the SRA hosted by the NCBI. The accession number for the SRA dataset is PRJNA1357863. Processed scRNA-seq data for peak and year 9.3 samples have been deposited in the Gene Expression Omnibus under the accession no. GSE311890. Source data are provided with this paper.

## Extended data

**Extended Data Table 1:** Long-term CART19 persistence

Patient ID	Time from CART19 infusion	CAR19 copies/μg DNA	B cells/μL
#5	5.8 years	0	130^#^
#6	9.3 years	0	312
#11	10.1 years	10.5	186
#14	8.6 years	262	<10
#17	10.1 years	267	<10
#21	9.3 years	1567	0
#33	7.8 years	0	109
#36*	7.0 years	64	353

CAR transgene copies were quantified by qPCR in the eight patients with samples available beyond 5 years after CART19 infusion. For each patient, time from infusion, CAR transgene copy number, and the closest available B-cell count are reported. **matched flow cytometry for phenotypical detection of CART19 was performed on the closest peripheral blood mononuclear sample available that was collected during year 6*. ^*#*^*Data were extrapolated based on the last available B-cell percentage evaluation (Month 24), as CART19 transgene copies were already 0 copies/μg DNA; estimation was derived starting from the lymphocyte count*.

## Code availability

Packages used in the analyses include: ggplot2 (v.3.5.2), ReactomePA (v.1.52.0), clusterProfiler (v.4.16.0), Nebulosa (v.1.18.0), Seurat (v.5), Cell Ranger (v.8.0.1), scRepertoire (v.2.5.1), scDblFinder v.1.22.0, tidyverse (v.2.0.0), entropy (v.1.3.2), ggalluvial (v. 0.12.5), pySCENIC (v.0.12.1), pandas (v.2.3.3), NumPy (v.1.23.5), SciPy (v.1.15.2), matplotlib (v.3.10.8), Matrix (v.1.7.4), glmnet (v.4.1-10), dplyr (v.1.1.4), pheatmap (v.1.0.12), AnnotationDbi (v.1.72.0) and org.Hs.eg.db (v.3.22.0). The code used for the analysis is available at https://github.com/EDCAKDC/GSE311890-CART-analysis.
