# Publisher Correction: Decade-long persistence of CD19 CAR T cells in B cell lymphomas

**DOI:** 10.1038/s41591-026-04668-0

**Published:** 2026-08-26

**Authors:** Luca Paruzzo, Emeline R. Chong, Elise A. Chong, Zhixuan Zheng, Puneeth Guruprasad, Daniel J. Landsburg, Sunita D. Nasta, Federico Stella, Ranjani Ramasubramanian, Vitor B. de Souza, Mohannad H. Alkhatib, Matthew Ho, Antonio Imparato, Gregory M. Chen, Don L. Siegel, Gabriela Plesa, Anlan Dai, Shane Mackey, Priyamvada Das, Peter Michener, Julie Jadlowsky, Frederic D. Bushman, Vrutti Patel, Alberto Carturan, Ellen B. Napier, Vanessa E. Gonzalez, Danuta Jarocha, Rong Xu, Bruce L. Levine, Patrizia Porazzi, Noelle Frey, David L. Porter, Jakub Svoboda, Joseph A. Fraietta, Carl H. June, Stephen J. Schuster, Marco Ruella

**Affiliations:** 1 Lymphoma Program, Abramson Cancer Center, University of Pennsylvania, Philadelphia, PA USA; 2 Center for Cellular Immunotherapies, University of Pennsylvania, Philadelphia, PA USA; 3 Department of Medicine, Division of Hematology/Oncology, Hospital of the University of Pennsylvania, Philadelphia, PA USA; 4 Department of Microbiology, Perelman School of Medicine, University of Pennsylvania, Philadelphia, PA USA; 5 Department of Pathology and Laboratory Medicine, Hospital of the University of Pennsylvania, Philadelphia, PA USA; 6 Center for Cell Therapy and Transplant, Philadelphia, PA USA

**Keywords:** Translational research, Cancer immunotherapy, B-cell lymphoma, Translational immunology

Correction to: *Nature Medicine* https://doi.org/10.1038/s41591-026-04578-1, published online 10 August 2026.

In the version of the article initially published, the *y*-axis label in Fig. 3c, g and the right panel of Fig. 5c was “log_10_(*P*_adj_)” but should have been “–log_10_(*P*_adj_)”. This correction has been made to the HTML and PDF versions of the article.

