# Supplementary material for: Decade-long persistence of CD19 CAR T cells in B cell lymphomas
Source: Nat Med. 2026 Aug 10;32(9):3276–87. doi: 10.1038/s41591-026-04578-1 (PMC13577893; doi:10.1038/s41591-026-04578-1)
Supplement: Supplementary file 1 [file 41591_2026_4578_MOESM1_ESM.pdf]

nature medicine

Supplementary information

https://doi.org/10.1038/s41591-026-04578-1

Decade-long persistence of CD19 CAR T cells in B cell lymphomas

In the format provided by the authors and unedited

Table of Contents

B-cell proportion at apheresis and correlation with long-term clinical response……………………2

B-cell reconstitution at 24 months according to the infusion product characteristics and the peak of expansion……………………………………………………………………………………………3

Infusion product characteristics and long-term clinical outcomes………………………………………4

DNMT3A and *TET2* expression in CAR$^+$ T compared to CAR$^-$T cells in patient #21 Y9.3. ……….5

Double-negative CAR T cells at peak expansion are not associated with overall response………6

Flow cytometry gating strategy for CAR T-cells enrichment for single-cell analysis……………….7

Z-score normalized average expression of lymphocyte clusters at year 9.3………………………..8

Year 9.3 CAR T transcriptional profile feature plots…………………………………………………….9

Year 9.3 CAR T transcriptional profile………………………………………………………………….10

Year 9.3 cell cycle phase in the lymphocyte population……………………………………………………………………………………………11

Year 9.3 CAR$^-$ versus CAR$^+$ T transcriptional profile…………………………………………………12

Z-score normalized average expression of CAR T clusters of the integrated object peak and year 9.3……………………………………………………………………………………………………… 13

Peak and year 9.3 CAR T transcriptional profile………………………………………………………14

Long-term CAR T-cells have a proliferative interferon-driven phenotype…………………………15

Flow cytometry dot plots from the commercial cohort showing negative CAR detection.............16

Distribution of patient #21, year 9.3 dominant clonotype (CSAGTWGTEAFF) at peak and long term……………………………………………………………………………………………………17

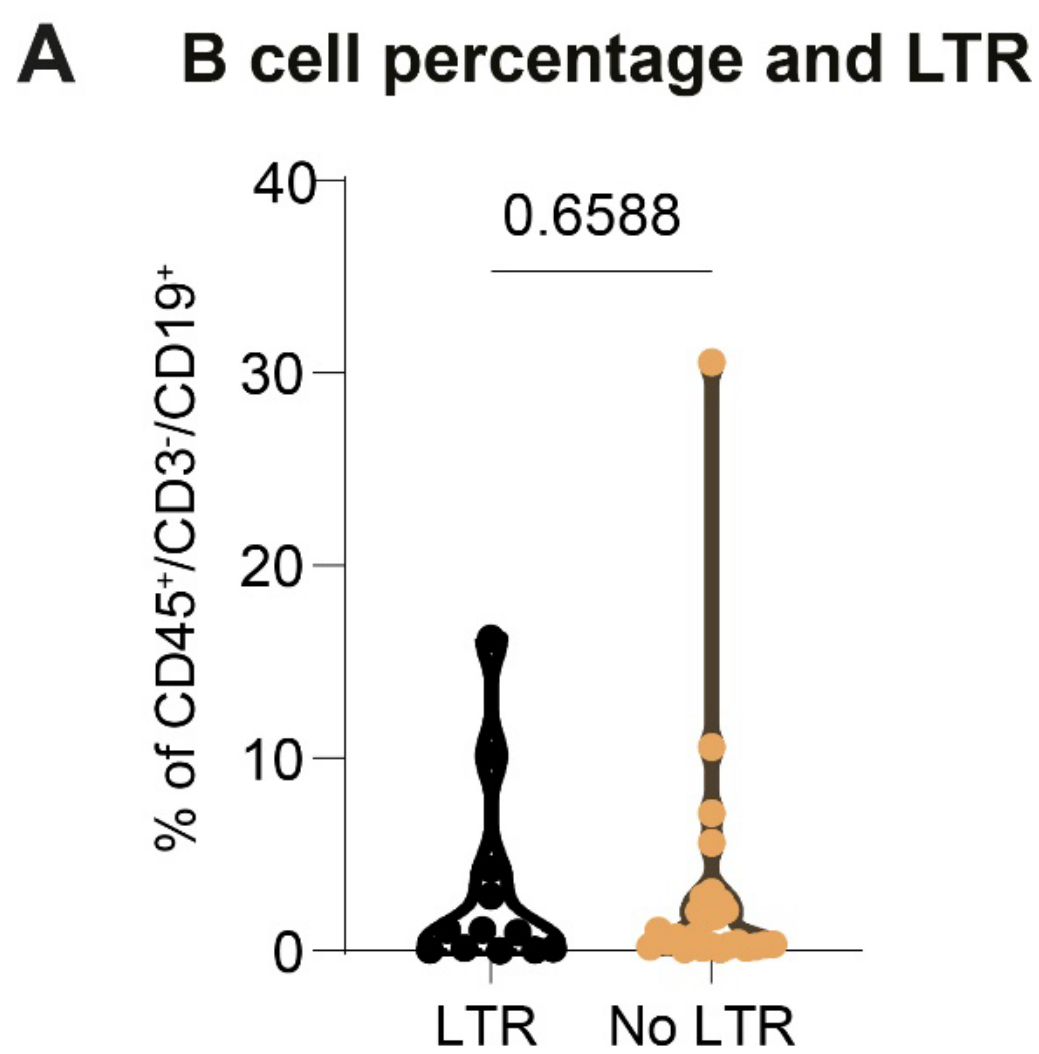

Supplementary Figure 1. B-cell proportion at apheresis and correlation with long-term clinical response. Violin plot representing the % of B cells ($CD45^+/CD3^-/CD19^+$) in PBMC at apheresis based on the long-term clinical response. Long-term clinical response (LTR) was defined as progression-free survival > 5.4 years, the time of the last relapse in the cohort. No differences were observed between LTR (n=12) and non-LTR (n=26). Comparison was performed using the unpaired Mann-Whitney test. Significance was set at 0.05

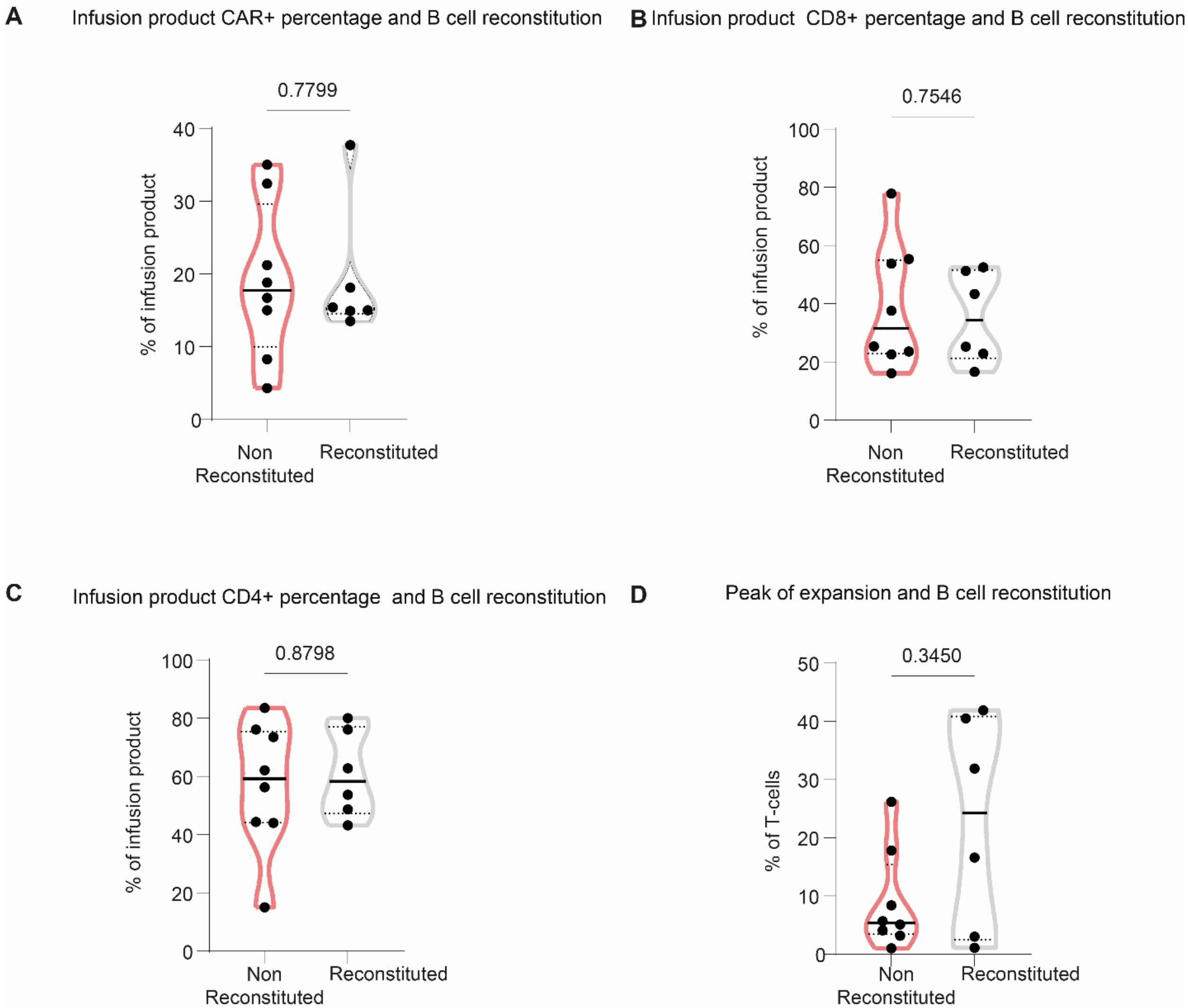

Supplementary Figure 2. B-cell reconstitution at 24 months according to the infusion product characteristics and the peak of expansion. (**A**) Percentage of CAR$^+$T cells in the infusion product of patients that reconstituted B cells (n=6) versus those without B-cell reconstitution at 24 months (n=8). (**B**) Percentage of CD8$^+$T cells in the infusion product of patients that reconstituted B cells (n=6) versus those without B-cell reconstitution at 24 months (n=8). (**C**) Percentage of CD4$^+$T cells in the infusion product of patients that reconstituted B cells (n=6) versus those without B-cell reconstitution at 24 months (n=8). (**D**) Percentage of CAR$^+$T cells out of T cells at the peak of expansion in patients that reconstituted B cells (n=6) vs not (n=8). B cells were considered reconstituted when they accounted for more than 2% of peripheral blood mononuclear cells. Groups were compared using a two-sided unpaired Mann-Whitney test. Statistical significance was defined as $p < 0.05$.

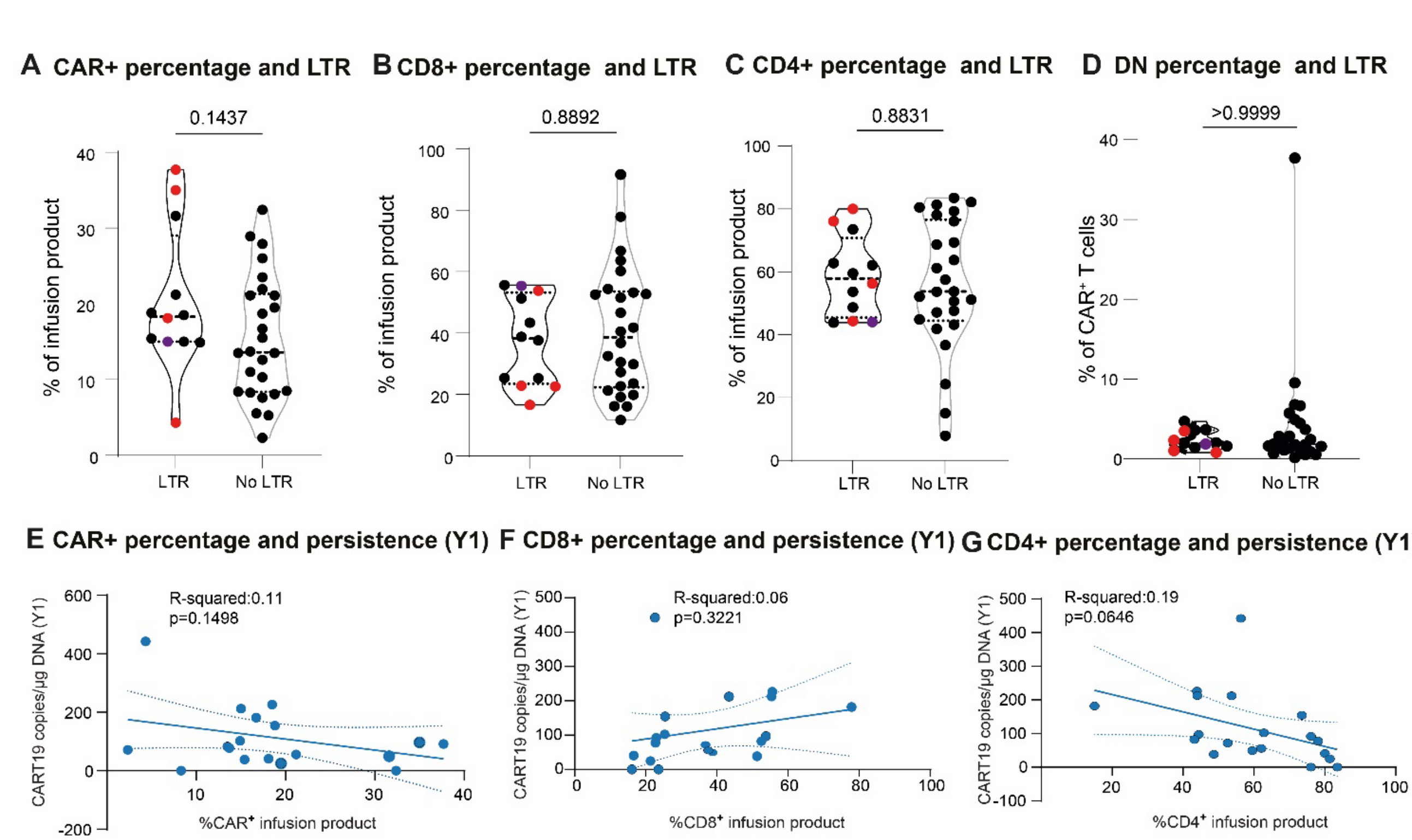

Supplementary Figure 3. Infusion product characteristics and long-term clinical outcomes. (**A**) Percentage of CAR⁺ T cells in the infusion product (total population, n=38) stratified by long-term response (LTR, defined as progression-free survival >5.4 years, corresponding to the time of last observed relapse, n=12). Red dots indicate patients with CAR T-cell persistence beyond year 5; patient #21, who has long-term persistence, is colored in purple. (**B**) Percentage of $CD8^+$ T cells in the infusion product (total population, n=38) according to LTR (n=12). Red dots indicate patients with CAR T-cell persistence beyond year 5; patient #21, who has long-term persistence, is colored in purple. (**C**) Percentage of $CD4^+$ T cells in the infusion product (total population, n=38) according to LTR (n=12). Red dots indicate patients with CAR T-cell persistence beyond year 5; patient #21, who has long-term persistence, is colored in purple. (**D**) Percentage of double-negative ($CD4^-CD8^-$) T cells within the CAR^+ compartment of the infusion product (total population, n=38) according to LTR (n=12). Red dots indicate patients with CAR T-cell persistence beyond year 5; patient #21, who has long-term persistence, is colored in purple. (**E**) Linear regression analysis of the percentage of CAR^+ T cells in the infusion product (n=19) versus CAR copies per microgram of DNA at 1 year. Only patients without disease progression at the 1-year landmark were included. (**F**) Linear regression analysis of the percentage of $CD8^+$ T cells in the infusion product (n=19) versus CAR copies per microgram of DNA at 1 year. (**G**) Linear regression analysis of the percentage of $CD4^+$ T cells in the infusion product (n=19) versus CAR copies per microgram of DNA at 1 year. Two side Unpaired Mann–Whitney tests were used for panels A-D. Linear regression was used in panel E-G. Statistical significance was defined as $p<0.05$.

A *DNMT3A* **and** *TET2* **expression in CAR- T cells compared to CAR+ T cells at Year 9.3 in patient #21**

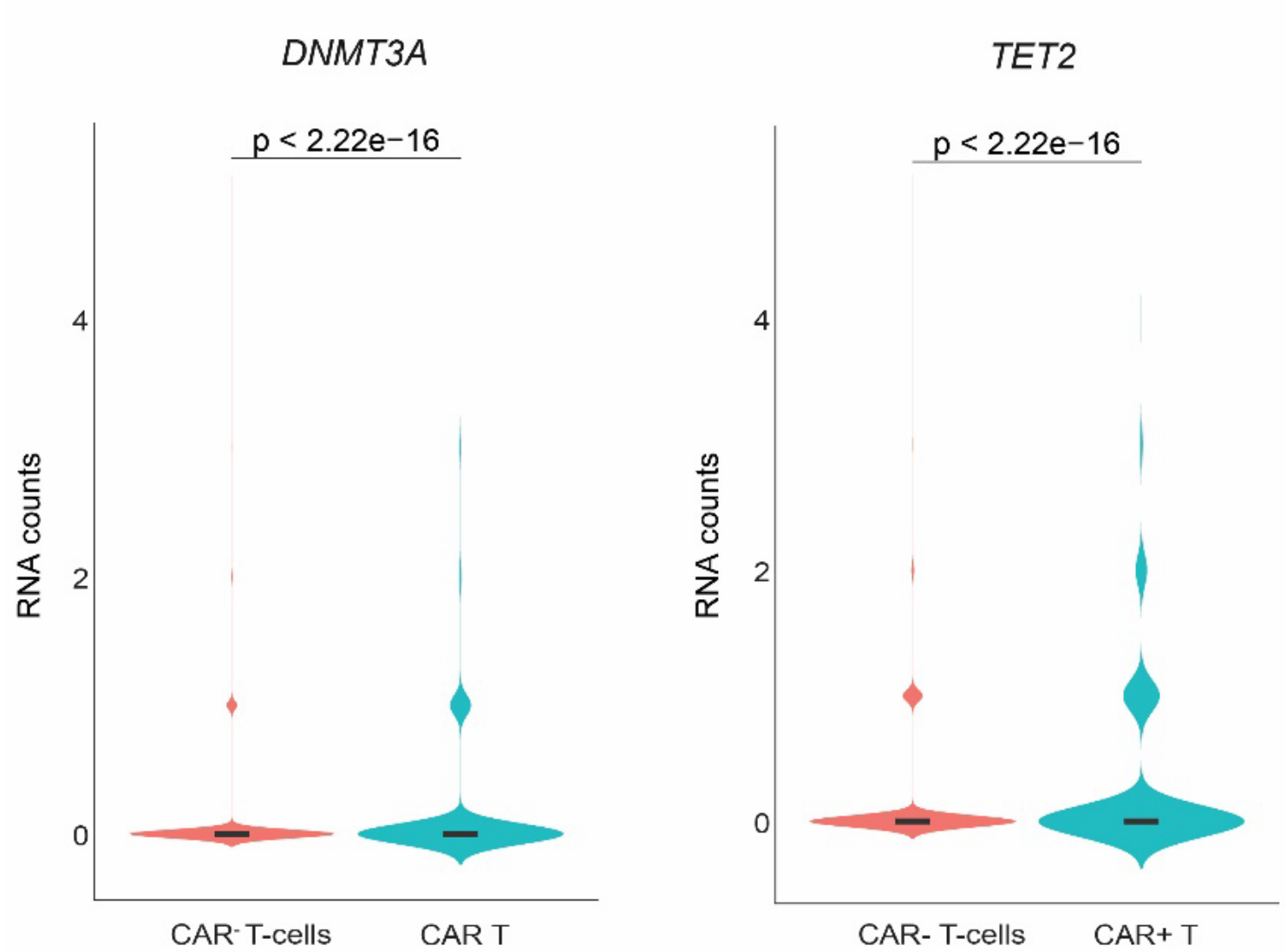

Gene	CAR- (%)	CAR+ (%)
DNMT3A expressing cells	5.4	10.4
TET2 expressing cells	9.9	19.9

Supplementary figure 4. ***DNMT3A*** **and** ***TET2*** **expression in CAR^{+} T compared to CAR^{-}T cells in patient #21 Y9.3.** (**A**) Violin plots showing RNA expression of *DNMT3A* and *TET2* in CAR^{+} T cells and CAR^{-}T cells at year 9.3 (left). Table summarizing the percentage of cells expressing *DNMT3A* and *TET2* (RNA count >0) in the CAR^{+} T and CAR^{-} compartments (right). P values were calculated using the two side Wilcoxon rank-sum test and adjusted for multiple testing using the Benjamini-Hochberg method. Exact p-values for DNMT3A is$1.25*10^{-16}$ while for TET2 is $2.11*10^{-35}$.

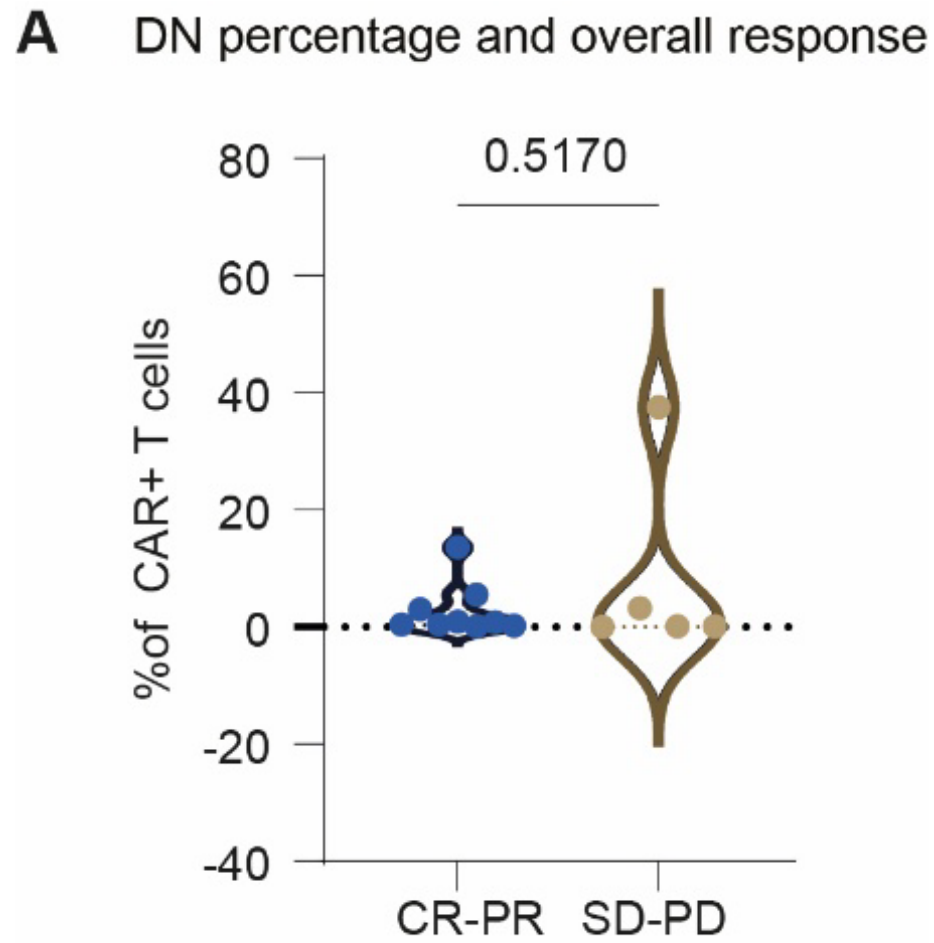

Supplementary Figure 5. Double-negative CAR T cells at peak expansion are not associated with overall response. Violin plot comparing the proportion of double-negative (DN; $CD4^{-}CD8^{-}$) CAR^{+} T cells at peak expansion (day 7-14, n=14) between patients achieving complete or partial response (CR/PR, n=9) and those with stable disease or progressive disease after CAR T-cell therapy (n=5). No significant differences were observed. Comparisons were performed using an unpaired two side Mann–Whitney, with statistical significance defined as $p<0.05$.

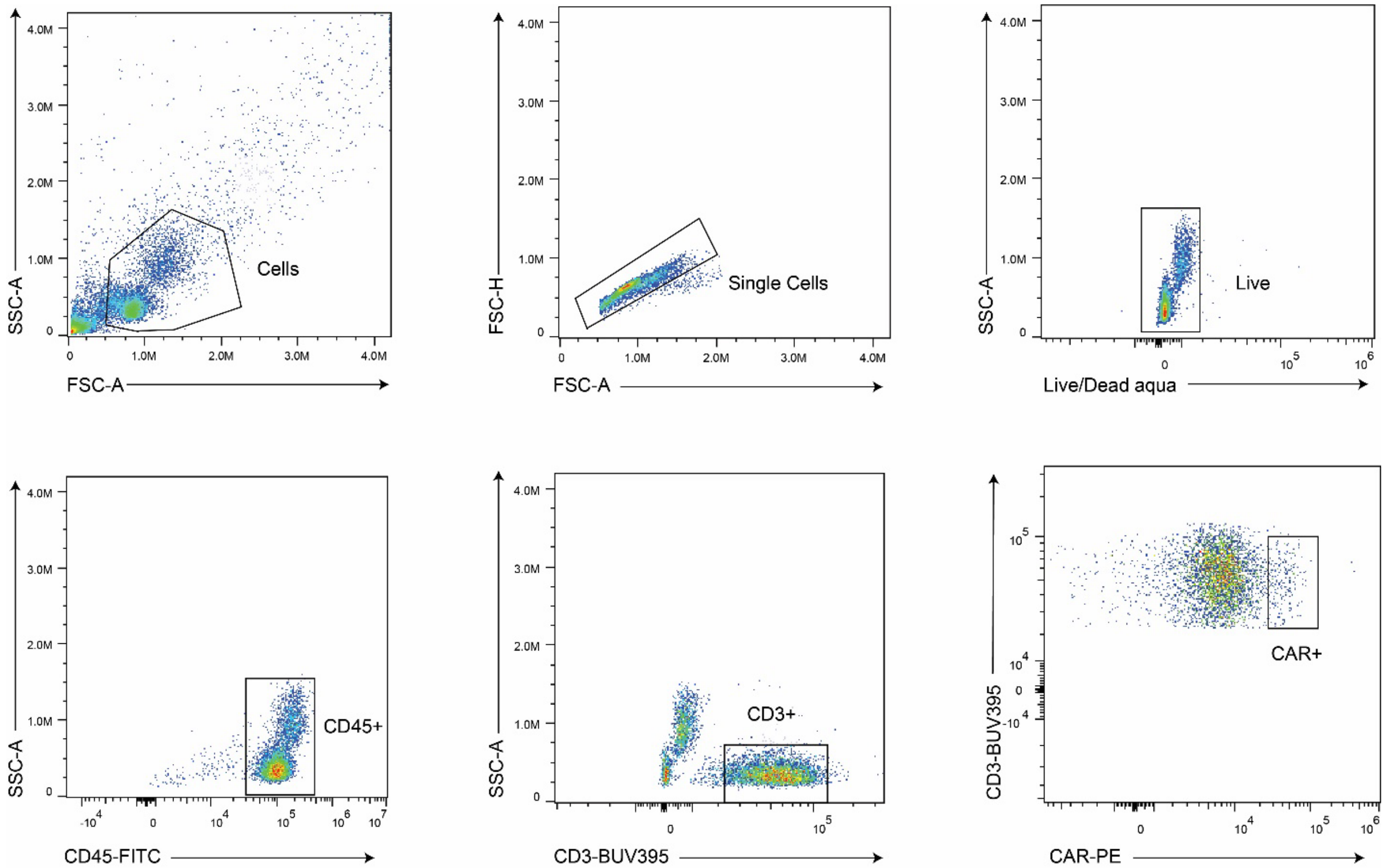

Supplementary Figure 6. Flow cytometry gating strategy for CAR T-cells enrichment for single-cell analysis. Flow cytometry gating strategy applied to isolate CAR T-cells before single-cell RNA sequencing at peak expansion and year 9.3. To preserve sufficient representation of this rare population in the sequencing analysis, 20,000 cells were recorded for the representative plot.

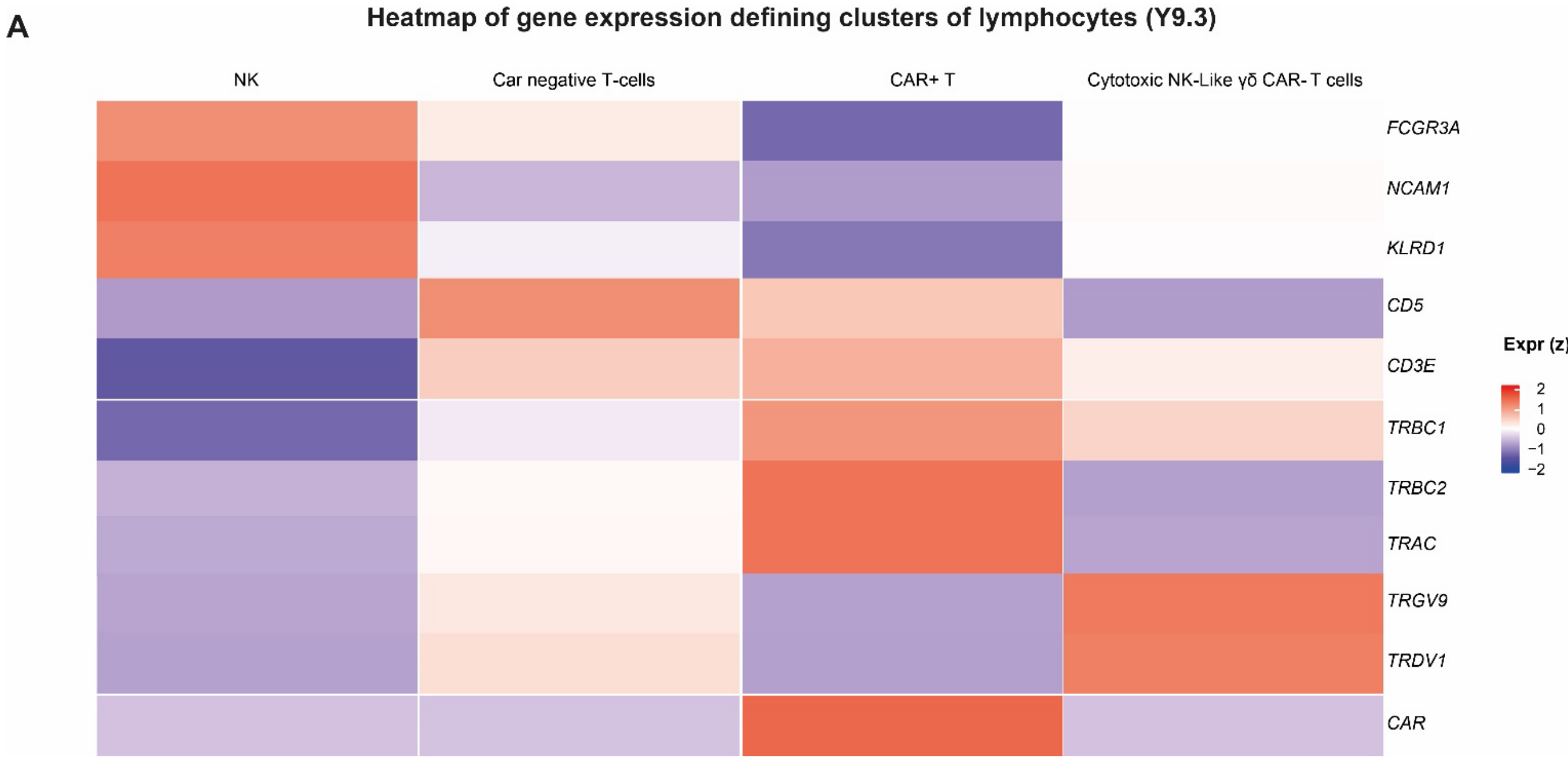

Supplementary Figure 7. Z-score normalized average expression of lymphocyte clusters at year 9.3. (**A**) Heatmap of gene expression defining clusters within the Uniform Manifold Approximation and Projection (UMAP) of year-9.3 lymphocytes. Shown is the average z-score-normalized expression of representative genes delineating each cluster.

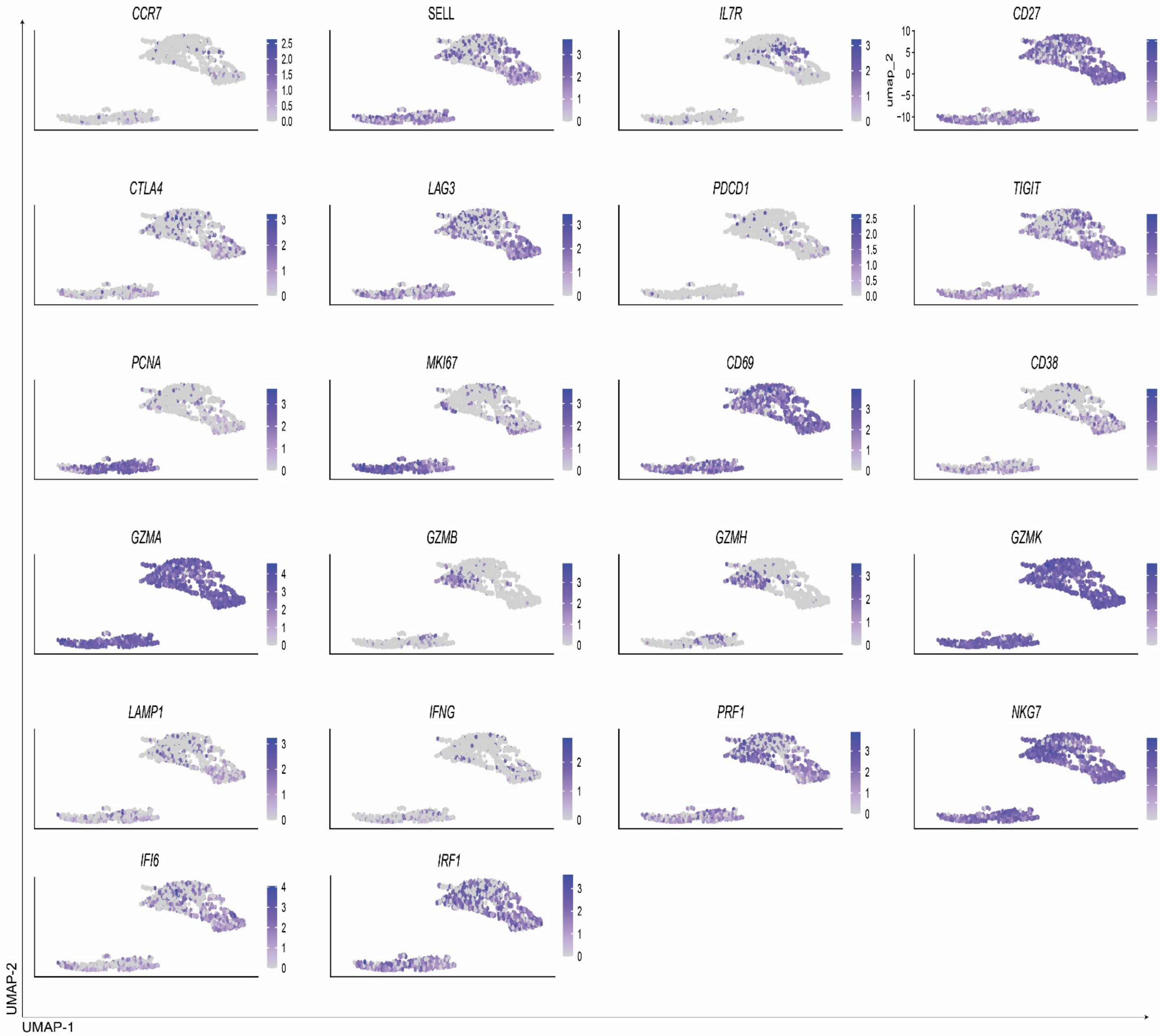

Supplementary Figure 8. Year 9.3 CAR T transcriptional profile feature plots. Selected gene feature plots describing the transcriptional profile of year 9.3 CAR T.

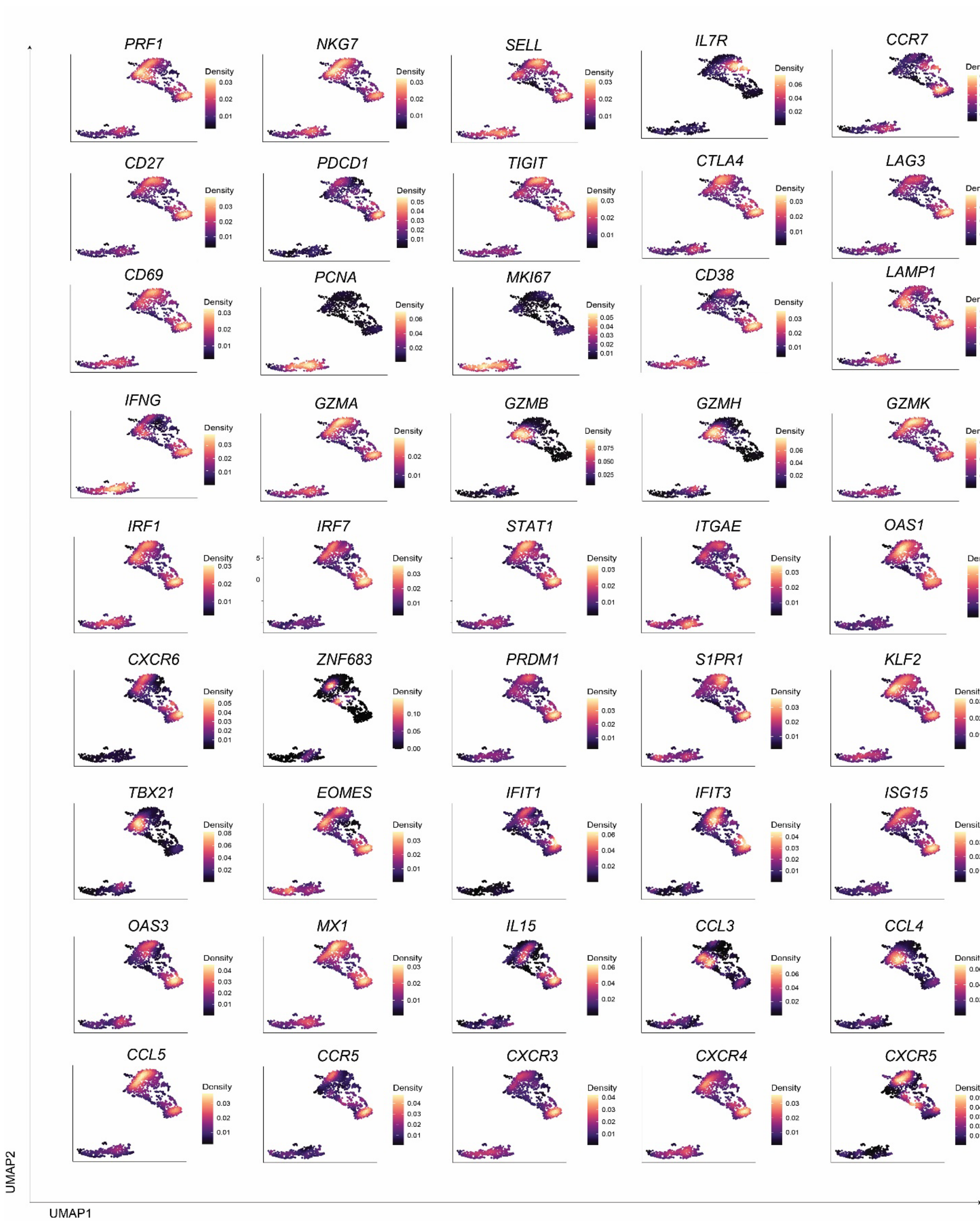

Supplementary Figure 9. Year 9.3 CAR T transcriptional profile. Selected gene density plots describing the transcriptional profile of year 9.3 CAR T.

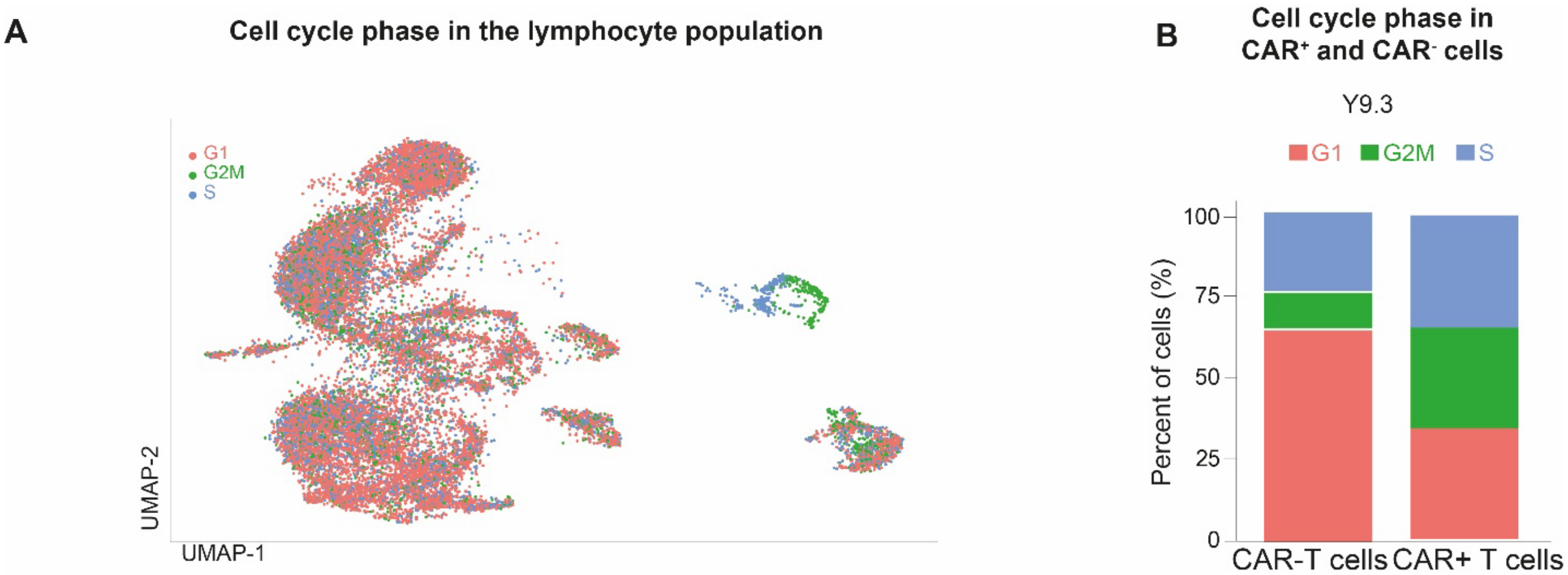

Supplementary Figure 10. Year 9.3 cell cycle phase in the lymphocyte population. (**A**) Cell cycle phase projected on Uniform Manifold Approximation and Projection (UMAP) of the lymphocyte population at Y 9.3. (**B**) Proportion of cells in each cell cycle phase in CAR^- versus CAR^+ T Cells at year 9.3 calculated in the lymphocyte population.

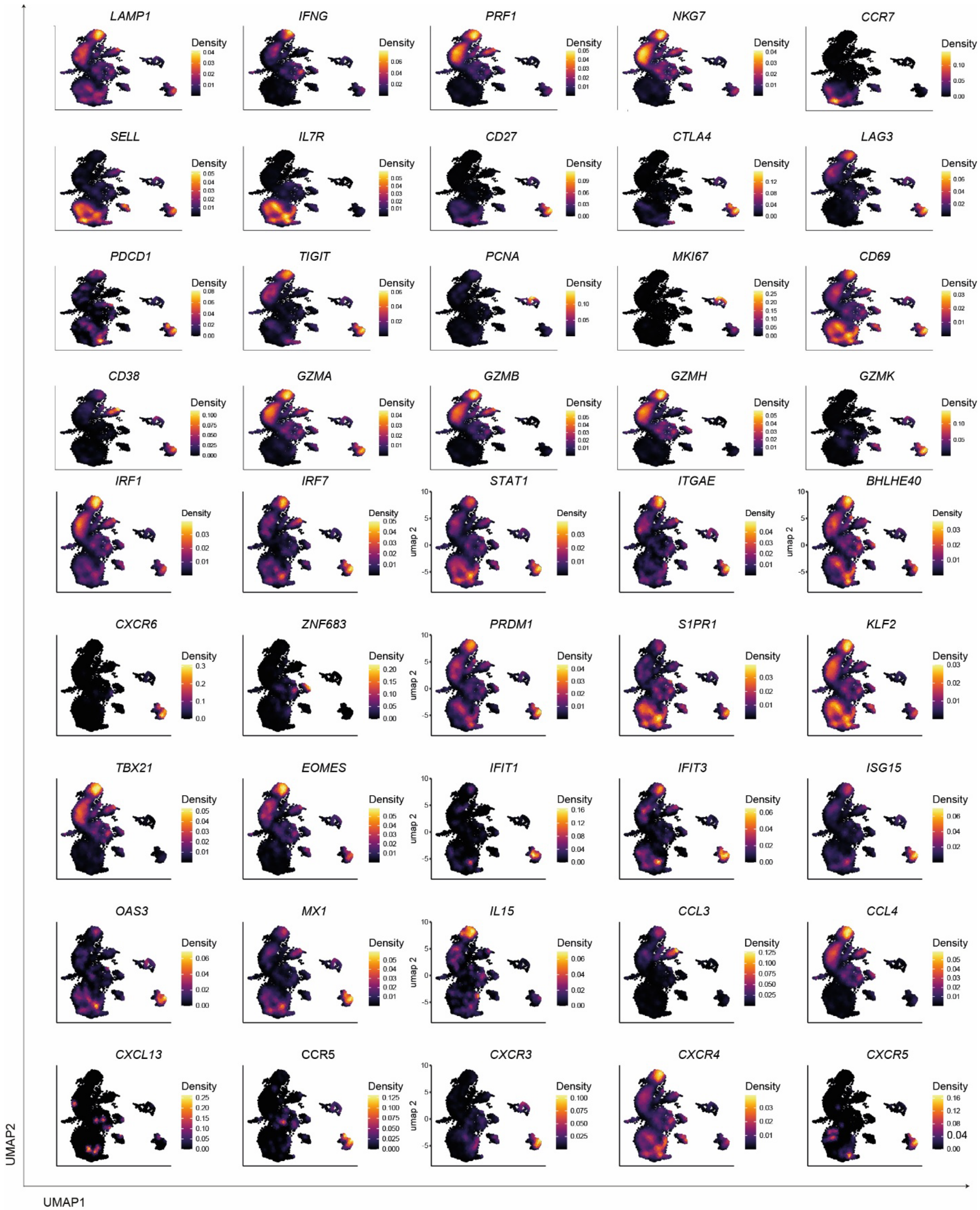

Supplementary Figure 11. Year 9.3 CAR$^-$ versus CAR$^+$ T transcriptional profile. Selected gene density plots describing the transcriptional profile of year 9.3 lymphocytes.

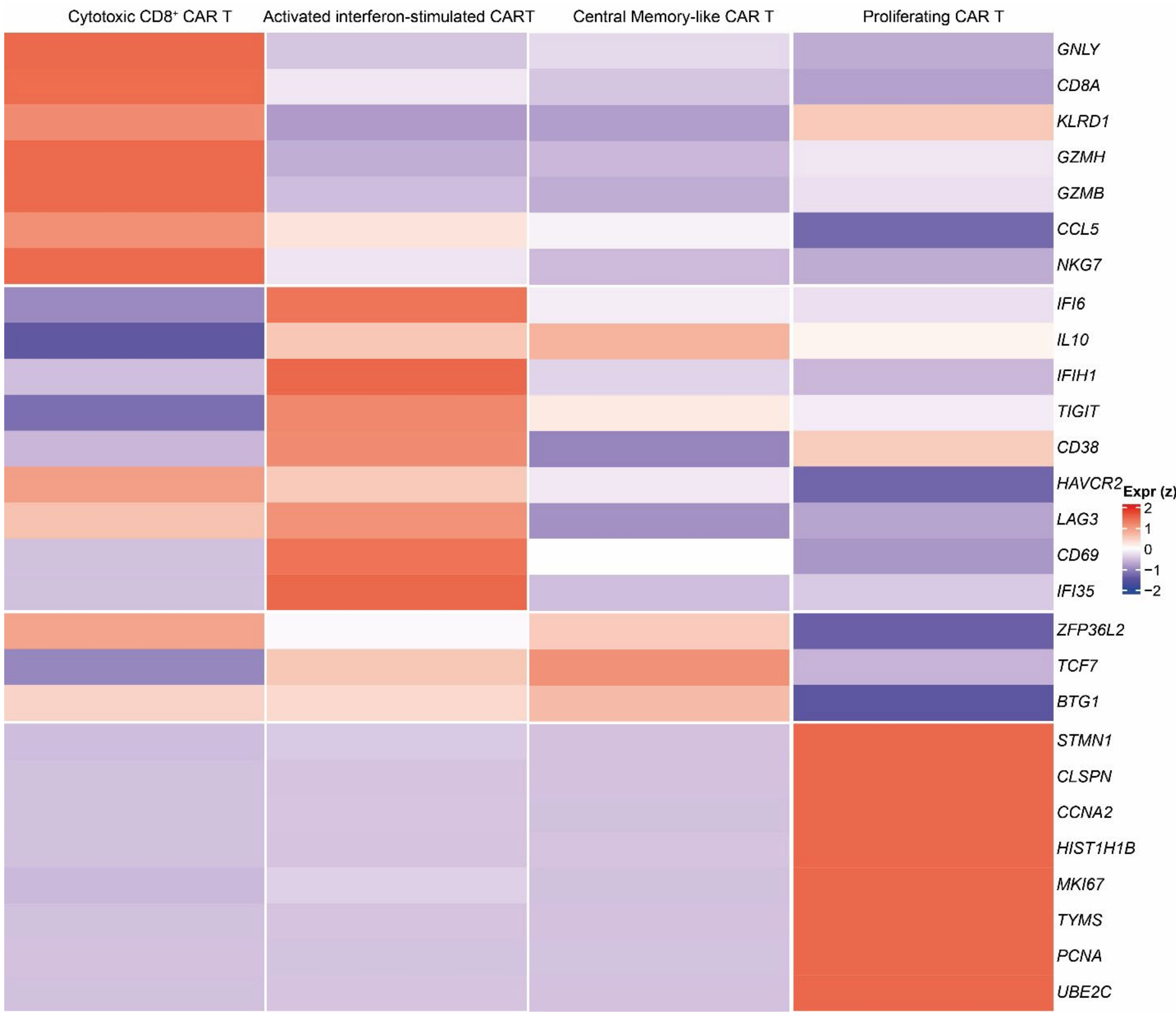

Supplementary Figure 12. Z-score normalized average expression of CAR T clusters of the integrated object peak and year 9.3. Heatmap of gene expression defining clusters within the integrated Uniform Manifold Approximation and Projection (UMAP) of peak and year-9.3 CAR T-cells. Shown is the average z-score-normalized expression of representative genes delineating each cluster.

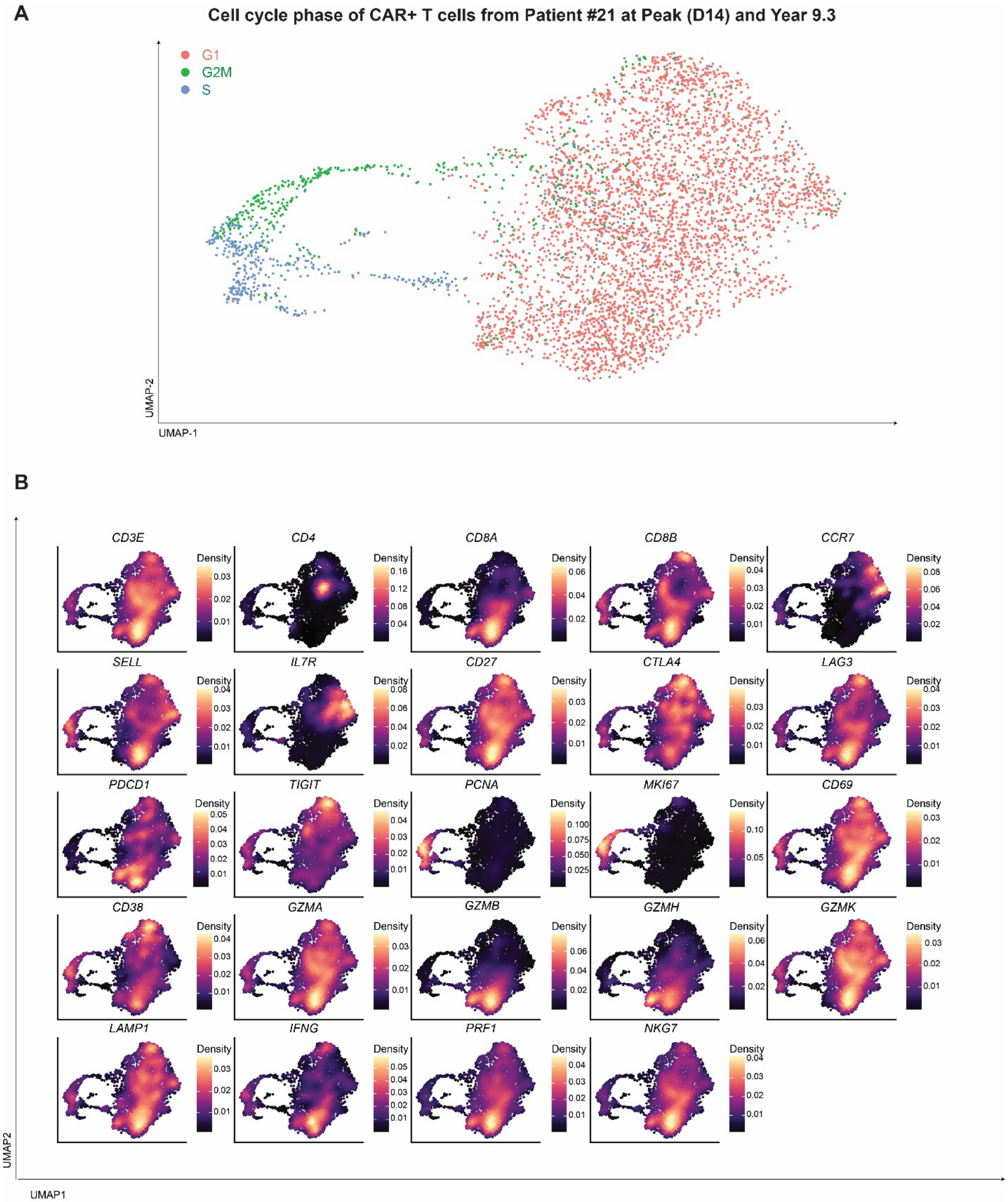

Supplementary Figure 13. Peak and year 9.3 CAR T transcriptional profile. (**A**) Cell cycle phase projected on Uniform Manifold Approximation and Projection (UMAP) of the integrated peak and year 9.3 CAR T. (**B**) Selected gene density plots describing the transcriptional profile of the integrated peak and year 9.3 CAR T.

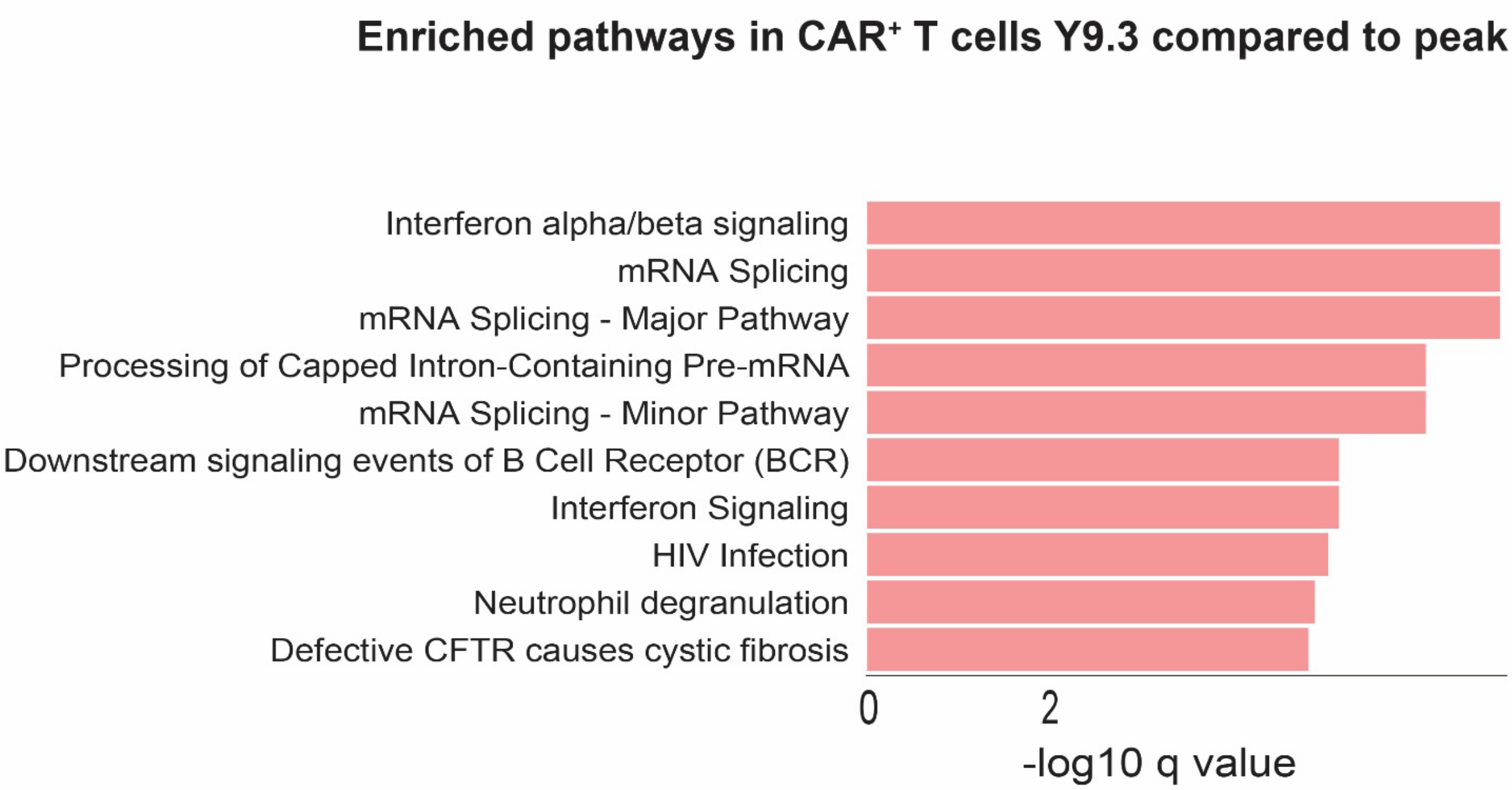

Supplementary Figure 14. Long-term CAR T-cells have a proliferative interferon-driven phenotype. Reactome pathways enrichment analysis comparing CAR T-cells in G1 phase at year 9.3 over the peak.

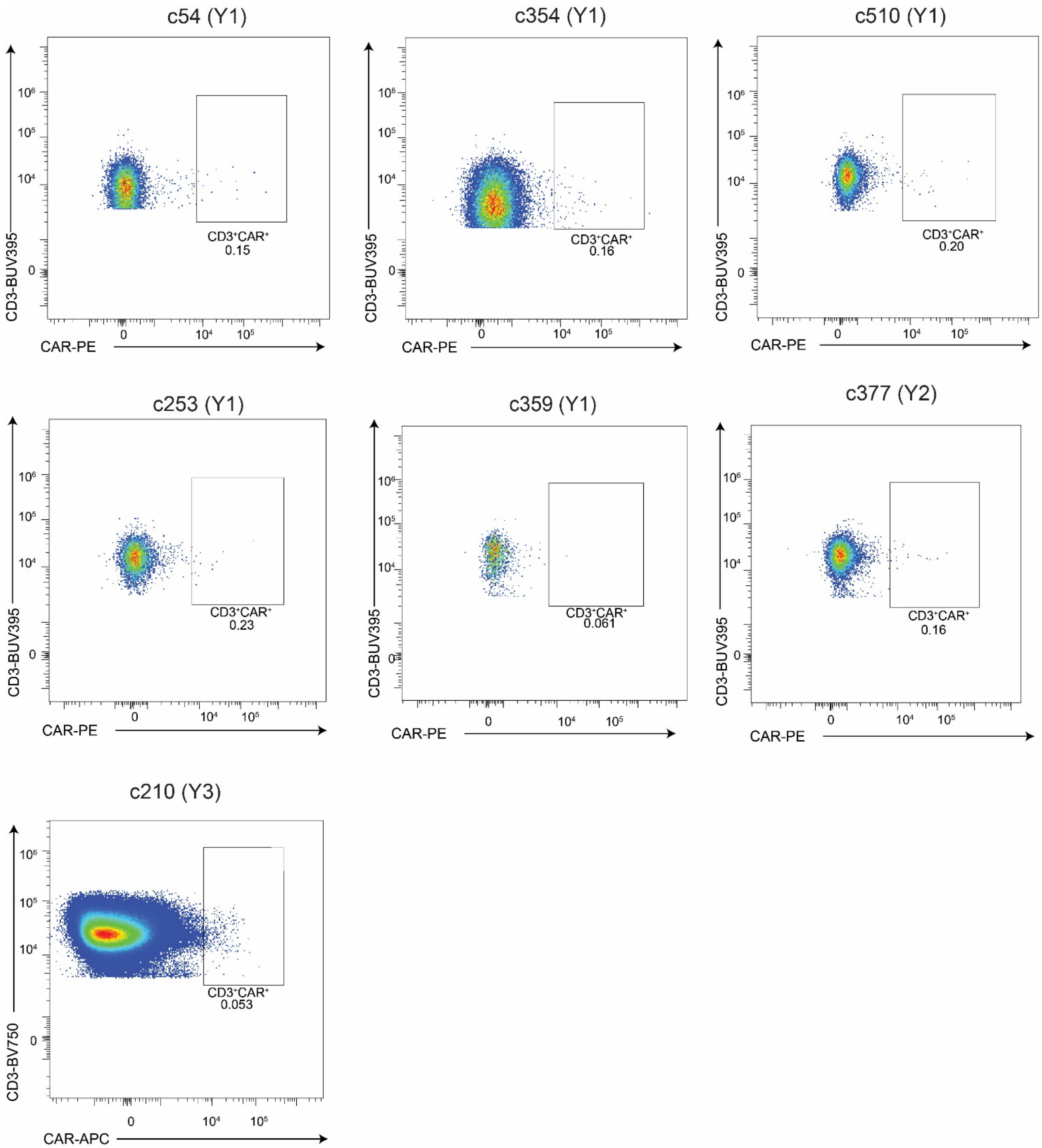

Supplementary Figure 15. Flow cytometry dot plots from the commercial cohort showing negative CAR detection. Negative flow cytometry findings from 7 additional commercial CART19-treated patients with samples collected between years 1 and 3 after infusion. CAR expression was assessed using an anti-FMC63 antibody, labeled with either APC or PE.

A

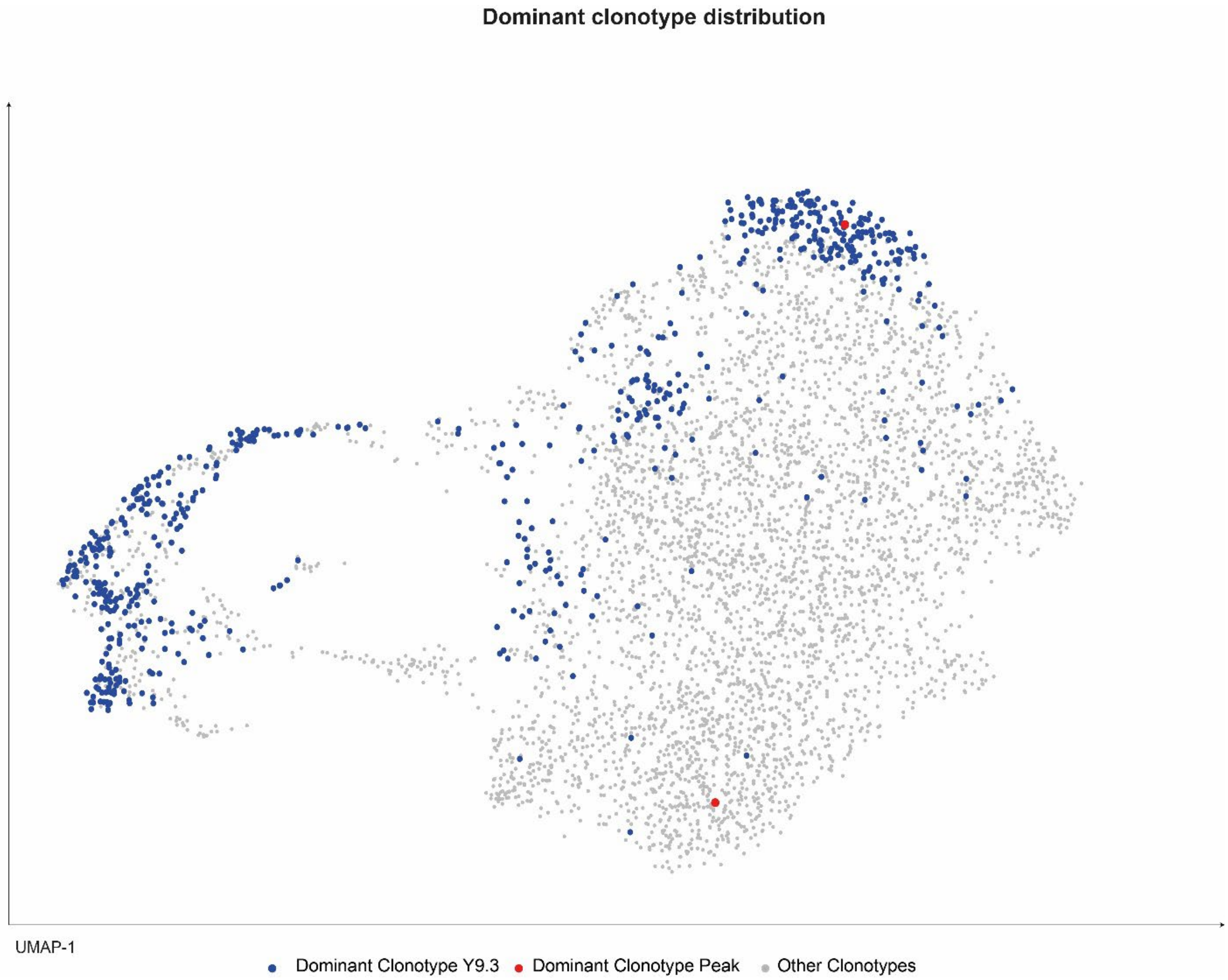

Supplementary Figure 16. Distribution of patient #21, year 9.3 dominant clonotype (CSAGTWGTEAFF) at peak and long term. This UMAP of the integrated CAR T cells from year 9.3 and peak time points shows the distribution of the dominant clonotype detected at year 9.3 (blue dots) and its corresponding cells at the peak time point (red dots). The dominant clonotype identified at year 9.3 was already present at the peak at a low level. At the peak time point, one cell mapped to the double-negative (DN) cluster and the other to the $CD8^+$ cluster.
