# Supplementary material for: Decade-long persistence of CD19 CAR T cells in B cell lymphomas
Source: Nat Med. 2026 Aug 10;32(9):3276–87. doi: 10.1038/s41591-026-04578-1 (PMC13577893; doi:10.1038/s41591-026-04578-1)
Supplement: Supplementary file 2 [file 41591_2026_4578_MOESM2_ESM.pdf]

nature portfolio

Corresponding author(s): Marco Ruella, Stephen J. Schuster

Last updated by author(s): 06/18/2026

Reporting Summary

Nature Portfolio wishes to improve the reproducibility of the work that we publish. This form provides structure for consistency and transparency in reporting. For further information on Nature Portfolio policies, see our Editorial Policies and the Editorial Policy Checklist.

Statistics

For all statistical analyses, confirm that the following items are present in the figure legend, table legend, main text, or Methods section.

n/a	Confirmed	
☐	☒	The exact sample size (*n*) for each experimental group/condition, given as a discrete number and unit of measurement
☐	☒	A statement on whether measurements were taken from distinct samples or whether the same sample was measured repeatedly
☐	☒	The statistical test(s) used AND whether they are one- or two-sided<br>*Only common tests should be described solely by name; describe more complex techniques in the Methods section.*
☒	☐	A description of all covariates tested
☐	☒	A description of any assumptions or corrections, such as tests of normality and adjustment for multiple comparisons
☐	☒	A full description of the statistical parameters including central tendency (e.g. means) or other basic estimates (e.g. regression coefficient) AND variation (e.g. standard deviation) or associated estimates of uncertainty (e.g. confidence intervals)
☐	☒	For null hypothesis testing, the test statistic (e.g. *F*, *t*, *r*) with confidence intervals, effect sizes, degrees of freedom and *P* value noted<br>*Give P values as exact values whenever suitable.*
☒	☐	For Bayesian analysis, information on the choice of priors and Markov chain Monte Carlo settings
☒	☐	For hierarchical and complex designs, identification of the appropriate level for tests and full reporting of outcomes
☐	☒	Estimates of effect sizes (e.g. Cohen's *d*, Pearson's *r*), indicating how they were calculated

Our web collection on statistics for biologists contains articles on many of the points above.

Software and code

Policy information about availability of computer code

Data collection	BD FACSDiva software (BD Bioscience,Franklin Lakes,NJ,USA), SpectroFlo (Cytek Bioscience, Fremont ,CA,USA), 10x Genomics Chromium iX single-cell platform (10x Genomics, Pleasanton, CA) ,4200 TapeStation and the High-sensitivity DNA 5000 ScreenTape (Agilent, Santa Clara, CA), Qubit Fluorometer 2.0 (Thermofisher, Waltham, MA),NextSeq 2000 (Illumina, San Diego, CA),CytoFlex LX Flow Cytometer (from Beckman Coulter, Indianapolis, Indiana, USA)
Data analysis	FlowJo™ v10.10 Software (FlowJo LLC), FlowSOM (2.12.0),R Studio V 12.1, R V4.3., ggplot2 (3.5.2), ReactomePA v1.52.0,clusterProfiler v4.16.0,Nebulosa v1.18.0,Seurat v5,Cell Ranger v8.0.1,scRepertoire v2.5.1, scDblFinder v1.22.0, tidyverse (2.0.0), entropy (1.3.2), ggalluvial (0.12.5),pySCENIC (v0.12.1), pandas (v2.3.3), NumPy (v1.23.5), SciPy (v1.15.2), matplotlib (v3.10.8),Matrix (v1.7.4), glmnet (v4.1-10), dplyr (v1.1.4), pheatmap (v1.0.12), AnnotationDbi (v1.72.0), and org.Hs.eg.db (v3.22.0), GraphPad Prism version 10.6.1

For manuscripts utilizing custom algorithms or software that are central to the research but not yet described in published literature, software must be made available to editors and reviewers. We strongly encourage code deposition in a community repository (e.g. GitHub). See the Nature Portfolio guidelines for submitting code & software for further information.

Data

Policy information about availability of data

All manuscripts must include a data availability statement. This statement should provide the following information, where applicable:

- Accession codes, unique identifiers, or web links for publicly available datasets
- A description of any restrictions on data availability
- For clinical datasets or third party data, please ensure that the statement adheres to our policy

All requests for raw and analyzed data and materials are reviewed within eight weeks by the University of Pennsylvania and the corresponding authors to determine whether they are subject to intellectual property or confidentiality obligations. Patient-related data not included in the paper may be subject to patient confidentiality. De-identified clinical data supporting the findings of this study are available from the corresponding authors upon reasonable request, subject to institutional review board approval, patient consent restrictions, and applicable institutional and federal regulations governing the sharing of human participant data. The email addresses for the corresponding authors are mruella@upenn.edu and Stephen.Schuster@pennmedicine.upenn.edu. Any data and materials that can be shared will be released via a material transfer agreement. The integration site sequencing data have been deposited in the Sequence Read Archive (SRA) hosted by the National Center for Biotechnology Information (NCBI). The accession number for the SRA dataset is PRJNA1357863. Processed scRNA-seq data for peak and year 9.3 samples have been deposited in the Gene Expression Omnibus under the accession code GSE311890. Source Data are provided with this paper.

Research involving human participants, their data, or biological material

Policy information about studies with human participants or human data. See also policy information about sex, gender (identity/presentation), and sexual orientation and race, ethnicity and racism.

Reporting on sex and gender	We described biological sex in the description of the case report patient
Reporting on race, ethnicity, or other socially relevant groupings	This is not reported in the study
Population characteristics	We assessed CAR-T persistence in the entire cohort of patients treated with CART19 on the NCT02030834 trial. Histologic diagnosis (LBCL vs FL) and type of lymphodepletion were used as a stratification variable to compare CAR-T cell expansion and long-term persistence across disease subtypes and lymphodepletion.
Recruitment	We evaluated all patients treated with CART19 on the NCT02030834 trial. The data cutoff for clinical follow-up was October 1, 2025. Samples used for validation were available through UPCC37418 (use of these samples was approved by the University of Pennsylvania ,IRB).
Ethics oversight	The study was approved by University of Pennsylvania Internal Review Board.

Note that full information on the approval of the study protocol must also be provided in the manuscript.

Field-specific reporting

Please select the one below that is the best fit for your research. If you are not sure, read the appropriate sections before making your selection.

☒ Life sciences ☐ Behavioural & social sciences ☐ Ecological, evolutionary & environmental sciences

For a reference copy of the document with all sections, see nature.com/documents/nr-reporting-summary-flat.pdf

Life sciences study design

All studies must disclose on these points even when the disclosure is negative.

Sample size	38 patients treated with CART19 on the NCT02030834 trial, for validation we selected B cell lymphoma patients with available samples in the Penn CART biobank (UPCC37418). All available samples and data were analyzed. No covariate were include in the ad prioram design of the study.
Data exclusions	No clinical trial related data were excluded
Replication	Correlative analyses were performed on patient samples; therefore, experiments such as single-cell RNA sequencing and multiparametric flow cytometry were each conducted once.
Randomization	Experiment were not randomized. All samples with sufficient material were processed and analyzed.
Blinding	Blinding was not relevant in this study as the purpose was to describe and characterize the long lived CART19 in B-cell lymphoma

Reporting for specific materials, systems and methods

We require information from authors about some types of materials, experimental systems and methods used in many studies. Here, indicate whether each material, system or method listed is relevant to your study. If you are not sure if a list item applies to your research, read the appropriate section before selecting a response.

Materials & experimental systems

n/a	Involved in the study
☐	☒ Antibodies
☒	☐ Eukaryotic cell lines
☒	☐ Palaeontology and archaeology
☒	☐ Animals and other organisms
☐	☒ Clinical data
☒	☐ Dual use research of concern
☒	☐ Plants

Methods

n/a	Involved in the study
☒	☐ ChIP-seq
☐	☒ Flow cytometry
☒	☐ MRI-based neuroimaging

Antibodies

Antibodies used

Multiparameter flowcytometry patient #21 and c#077 (See also Suppl table 10)
Markers Fluorochrome Manufacturer Company Manufacturer Cat # Clone
CD45RA BUV395 BD Biosciences 740298 HI100
Live/Dead BUV LIVE/DEAD™ Blue Invitrogen L34962
CD16 BUV496 BD Biosciences 612944 3G8
CD14 BUV563 BD Biosciences 741360 M5E2
TIM3 BUV615 BD Biosciences 752363 7D3
CD28 BUV737 BD Biosciences 612815 CD28.2
CD8 BUV805 BD Biosciences 612889 SK1
PD-1 BV421 Biolegend 329920 EH12.2H7
CD123 SB436 Invitrogen 62-1239-42 6H6
CD19 BV480 BD Biosciences 746457 HIB19
CD27 BV510 Biolegend 302836 O323
CD33 BV570 Biolegend 303417 WM53
CD56 BV605 Biolegend 318334 HCD56
HLA-DR BV711 BioLegend 900003908 L243
CD3 BV750 BD Biosciences 747058 SK7
CD4 BV785 Biolegend 317442 OKT4
EOMES FITC eBioscience 11-4877-42 WD1928
CD95 BB700 BD Biosciences 566542 DX2
TOX PE Miltenyi Biotec 130-120-716 REA473
CCR7 PE-CF594 BD Biosciences 630003 150503
CTLA-4 PE-Cy5 BD Bioscience 555854 BNI3
LAG-3 PE-Cy7 Invitrogen 25-2239-42 3DS223H
CAR19 (anti-ID CD19scFv protein) APC (conjugated in TSCL) Cooper Lab 136.20.1
CD34 AF647 Biolegend 343508 581
Ki67 AF700 BD Bioscience 561277 B56
Granzyme B APC-Fire 750 Biolegend 372210 QA16A02
CD45 APC-Fire810 Biolegend 304075 HI30

Flowcytometry for CAR evaluation in the validation cohort used the following antibody:
ViaKrome 808 Fixable Viability Dye (Beckman; Cat# C36628) or Live/Dead BUV LIVE/DEAD™ (Blue Invitrogen L34962).
anti-CD3 BUV395 (Clone UCHT1, BD horizon,563546) or anti-CD3 BV750 (clone SK7 BD horizon, 747058)) ,
anti-CD14 Pacific Blue 525 (Clone – RMO52,B00846) or BUV563 (Clone-M5E2,BD bioscience,741360),
anti-CAR (anti-FMC63- PE (novartis) or APC (Cooper Lab))

Validation

See methods section and supplementary table 10 with the reported manufacturer company to access the provider company with the specific reference for validation.

Clinical data

Policy information about clinical studies
All manuscripts should comply with the ICMJE guidelines for publication of clinical research and a completed CONSORT checklist must be included with all submissions.

Clinical trial registration

NCT02030834 trial. This analysis is reporting on long-term CART19 persistence. The study was approved by Internal Review Board.

Study protocol	Long-term CAR-T19 persistence was analyzed in patients treated with CD19-directed CAR T-cell therapy on the NCT02030834 clinical trial at the University of Pennsylvania. Study was approved by university of Pennsylvania Internal review board.
Data collection	2014-2025 at the university of Pennsylvania
Outcomes	Long term CART19 persistence and phenotype in the whole cohort and by histology and lymphodepletion. This is not a clinical trial. There were no primary or secondary outcomes reported in this manuscript.

Plants

Seed stocks	Not applicable
Novel plant genotypes	Not applicable
Authentication	Not applicable

Flow Cytometry

Plots

Confirm that:

☒ The axis labels state the marker and fluorochrome used (e.g. CD4-FITC).

☒ The axis scales are clearly visible. Include numbers along axes only for bottom left plot of group (a 'group' is an analysis of identical markers).

☒ All plots are contour plots with outliers or pseudocolor plots.

☒ A numerical value for number of cells or percentage (with statistics) is provided.

Methodology

Sample preparation	See methods
Instrument	Cytek Aurora flow cytometer (Bethesda, MD, USA) equipped with a UV laser (355nm), a violet (405 nm), blue (488 nm), green (560 nm), and a red (640 nm) laser and CytoFlex LX Flow Cytometer (from Beckman Coulter, Indianapolis, Indiana, USA)
Software	FlowSOM, FlowJo
Cell population abundance	In each graph is reported the abundance of each cell subgroups.
Gating strategy	For T-cell analyses, cells were sequentially gated as lymphocytes → singlets → live cells → $CD3^{+}/CD14^{-}$. The full gating strategy used for cell sorting is shown in Supplementary Figure 1. Gating strategies for all T-cell subsets of interest are provided in the main figures and in the extended data figures.

☒ Tick this box to confirm that a figure exemplifying the gating strategy is provided in the Supplementary Information.
